# Engineering catalyst–support interactions in cobalt phthalocyanine for enhanced electrocatalytic CO_2_ reduction: the role of graphene-skinned Al_2_O_3_†

**DOI:** 10.1039/d5sc02616d

**Published:** 2025-05-22

**Authors:** Qianqian Bai, Bingyun Ma, Le Wei, Mutian Ma, Zhangyi Zheng, Wei Hua, Zhenyang Jiao, Min Wang, Huihong Yuan, Zhihe Wei, Tao Cheng, Xiaoxing Ke, Jun Zhong, Fenglei Lyu, Zhao Deng, Yang Peng

**Affiliations:** a Soochow Institute of Energy and Material Innovations, College of Energy, Jiangsu Key Laboratory for Advanced Negative Carbon Technologies, Soochow University Suzhou 215006 China fllv@suda.edu.cn zdeng@suda.edu.cn ypeng@suda.edu.cn; b Institute of Functional Nano & Soft Materials (FUNSOM), Soochow University Suzhou 215123 China; c Beijing Key Laboratory of Microstructure and Properties of Solids, College of Materials Science and Engineering, Beijing University of Technology Beijing 100124 China

## Abstract

Electrocatalytic CO_2_ reduction (eCO_2_R) driven by renewable electricity holds great promise to mitigate anthropogenic CO_2_ emissions. In this study, we engineer cobalt phthalocyanine (CoPc) supported on graphene-skinned Al_2_O_3_ nanosheets (CoPc/Al_2_O_3_@C) to enhance CO_2_-to-CO conversion. The strong π–π stacking between the CoPc macrocycle and interlayer graphene, coupled with electronic repulsion between the Co^2+^ center and Al_2_O_3_, induces a structural distortion in CoPc, raising the energy level of the d_*z*^2^_ orbital. This structural perturbation facilitates CO_2_ activation, shifts the rate-determining step, and thereby substantially accelerates the overall eCO_2_R kinetics. The optimal catalyst demonstrates a near-unity CO faradaic efficiency (FE_CO_) across a wide current range, achieving a high CO partial current density of 388 mA cm^−2^ with an exceptional turnover frequency (TOF) of 43 s^−1^, in addition to prolonged operational stability in a membrane electrode assembly (MEA). This work, by leveraging the vectorial interactions between molecular moieties and the substrate to reshape the macrocyclic structure and realign the orbital energies of CoPc, offers new insights into the design of efficient electrocatalysts for eCO_2_R.

## Abstract 2

This work, by leveraging the interactions between the molecule and the substrate to reshape the macrocyclic structure and realign the orbital energies of metal phthalocyanine, offers new insights into design of electrocatalysts for CO_2_ reduction.

## Graphical abstract

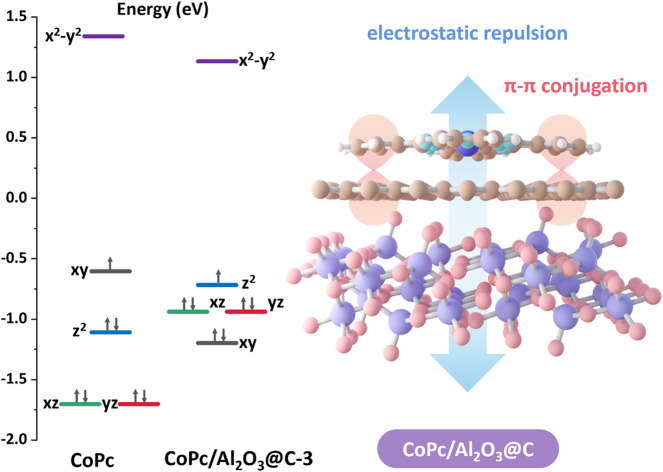

## Introduction

Electrocatalytic CO_2_ reduction (eCO_2_R) has emerged as a promising strategy for converting CO_2_ from flue gas into value-added chemicals and fuels using intermittent renewable energy sources.^1–5^ While significant advancements have been achieved in producing multi-electron transfer products and complex hydrocarbons through electrochemical conversion,^6–13^ carbon monoxide (CO) remains one of the most economically viable products for industrial-scale implementation. This preference stems from its advantages as the simplest two-electron reduction product, including low kinetic barriers, high selectivity, and seamless integration potential with existing Fischer–Tropsch processes.^14–18^

Metal phthalocyanines, particularly cobalt phthalocyanine (CoPc) and nickel phthalocyanine (NiPc) variants, have attracted considerable attention as model heterogeneous catalysts for CO_2_-to-CO conversion. These macrocyclic compounds feature a central M–N_4_ coordination site embedded within an 18π–electron conjugated framework, offering distinct advantages in terms of well-defined electronic structures, superior chemical stability, and cost-effectiveness.^16,19,20^ However, the sluggish CO_2_ activation, severe agglomeration caused by intermolecular π–π stacking, and intrinsically low electronic conductivity undermine their catalytic activity, leading to high overpotentials and low current densities.^21,22^ This necessitates the utilization of a conductive support to load the molecular catalysts, for not only improving the conductivity but also enhancing the dispersity.^23–25^

To promote the intrinsic catalytic activity of metal phthalocyanines, particularly in CO_2_ activation, extensive research efforts have focused on two primary modification strategies: structural modification of the phthalocyanine macrocycle through substituent groups (such as –CN, –F, –OC_8_H_17_, –OC_2_H_5_, –NH_2_, and –N(CH_3_)^+^)^16,21,26–29^ and axial coordination engineering at metal centers (such as pyridine).^30,31^ The former approach primarily aims to tailor the electronic environment of the metal center through strategic incorporation of electron-donating/withdrawing groups, while the latter seeks to disrupt molecular symmetry and modulate the energy distribution of 3d orbitals. During eCO_2_R, the initial CO_2_ activation on metal phthalocyanines is generally considered to involve one electron transfer from the d_*z*^2^_ orbital of the transition metal center to the unoccupied π* orbital of CO_2_, which is hindered by the high energy barrier.^31,32^ Therefore, the first activation step is often regarded as the rate-determining step (RDS) in converting CO_2_ to CO, which can be facilitated by raising the orbital level of d_*z*^2^_.

An alternative strategy to enhance the catalytic activity of metal phthalocyanines involves leveraging strong catalyst–support interactions. While carbonaceous materials such as graphene, carbon nanotubes (CNTs), and carbon quantum dots with tunable curvature have demonstrated effectiveness in modulating π–π interactions and improving molecular dispersity,^23,33–36^ nanostructured metal oxides have also been employed to fine-tune the electronic structure of the metal centers.^37,38^ For instance, Reisner *et al.* immobilized cobalt phthalocyanine using four phosphonic acid groups (CoPcP) onto TiO_2_ and achieved high performance for CO_2_ reduction to CO.^39^ The intimate contact between CoPc and TiO_2_ not only offers a stable support but also modulates the electronic properties of the metal centers, thereby enhancing the catalytic efficiency. In our previous study, Mg(OH)_2_, serving as Lewis acid sites, enabled the polarization of CO_2_ molecules adsorbed at the metal centers of CoPc, significantly enhancing CO selectivity at reduced overpotentials.^40^ Despite the notable benefits of oxide supports in enhancing the eCO_2_R performance, the turnover frequency (TOF) and partial current density of the reduction product remain constrained by limited electronic conductivity and poor molecular dispersity.

In this study, to harness both the π–π interaction and 3d orbital modulation between the molecular catalyst and underlying support, graphene-skinned Al_2_O_3_ nanosheets are fabricated to support CoPc molecules (CoPc/Al_2_O_3_@C) for electrochemical CO_2_ to CO conversion. Owing to the strong π–π stacking between the macrocyclic Pc ring and the graphene interlayer, coupled with the opposite electronic repulsion between the divalent metal center and the underlying Al_2_O_3_ substrate, the CoPc molecule bends down, deviating from its square-planar configuration with a distorted *D*_4h_ symmetry and realigning the Co 3d orbitals with the raised energy level of d_*z*^2^_. As a result, CoPc/Al_2_O_3_@C-3 with an optimal graphene thickness achieved a near-unity CO faradaic efficiency (FE_CO_) across a wide current range, achieving a high partial current density of 388 mA cm^−2^ with an exceptional TOF of 43 s^−1^, as well as prolonged operational stability in a membrane electrode assembly (MEA). This work underscores the importance of engineering molecule–support interactions to reshape the macrocyclic structure and realign the orbitals of metal centers, thereby enhancing eCO_2_R kinetics.

## Results and discussion

### Catalyst preparation and structural characterization

**Fig. 1:**
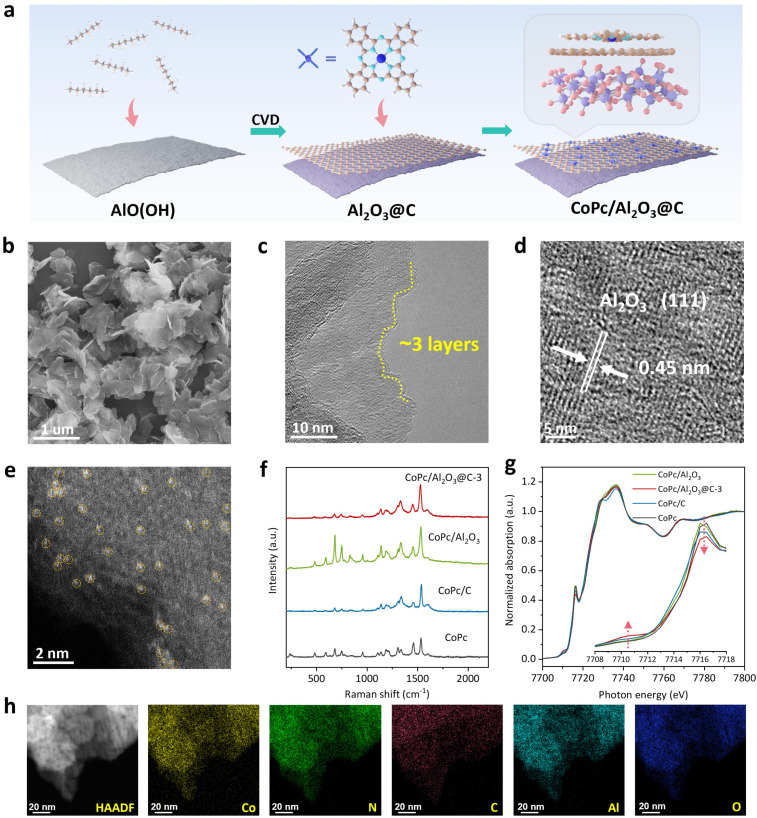
Fabrication and structural characterization of CoPc/Al_2_O_3_@C-*x*. (a) Schematic illustration of the synthetic process. (b) SEM, (c) TEM, (d) HR-TEM, and (e) aberration-corrected HAADF-STEM images of CoPc/Al_2_O_3_@C-3. (f) Raman and (g) Co K-edge XANES spectra of CoPc/Al_2_O_3_@C-3, CoPc/Al_2_O_3_, CoPc/C and CoPc (inset: the amplified edge absorption features). (h) HAADF-STEM image and the corresponding EDS elemental mapping images of N, Al, Co, C and O of CoPc/Al_2_O_3_@C-3.

CoPc molecules supported on graphene-skinned Al_2_O_3_ nanosheets, denoted as CoPc/Al_2_O_3_@C-*x* (*x* refers to the approximate layer number of graphene skins), were prepared by a sequential chemical vapor deposition (CVD) and molecular self-assembly approach (Fig. 1a), which is detailed in the Experimental section. Briefly, AlO(OH) nanosheets were first synthesized and used as both the template and catalyst for subsequent CVD deposition of the graphene overlayer. During the CVD process, AlO(OH) was thermally dehydrated into γ-Al_2_O_3_, serving as a Lewis acid to catalyze the graphitic transformation of hexane (used as the carbon source) on its surface (Fig. S1 and S2†). The thickness of the graphene overlayer (*x*) can be fine-tuned by varying the hexane amount, which will be detailed later.

Here, CoPc/Al_2_O_3_@C-3 is taken as an example to illustrate the microstructure and the intricate molecule–support interactions. Scanning electron microscopy (SEM) revealed that CoPc/Al_2_O_3_@C-3 exhibits a 2D lamellar morphology of nanosheets (Fig. 1b). While confirming the ultrathin nature of CoPc/Al_2_O_3_@C-3, transmission electron microscopy (TEM) images distinctly reveal lattice fringes with a *d*-spacing of approximately 0.35 nm, indicative of the crystalline graphene layer (Fig. 1c). Zooming into the nanosheet surface, lattice spacings of 0.45 nm corresponding to the (111) plane of γ-Al_2_O_3_ are clearly discernible (Fig. 1d). In the aberration-corrected high-angle annular dark-field scanning transmission electron microscopy (AC-HAADF-STEM) image in Fig. 1e, single atomic Co sites with high *Z*-contrast are distinguishable and highlighted by yellow dotted circles. This observation strongly suggests the molecular-level dispersion of CoPc on the surface of Al_2_O_3_@C-3, which is further verified by the energy-dispersive X-ray spectroscopy (EDS) elemental mapping images showing the uniform distribution of Co, N, C, Al and O elements across CoPc/Al_2_O_3_@C-3 (Fig. 1h). The coexistence and overlapping signals of these elements strongly support the successful construction of the CoPc/Al_2_O_3_@C heterojunction.

For comparative control studies, two additional samples were synthesized: CoPc/Al_2_O_3_, prepared by directly loading CoPc molecules onto the γ-Al_2_O_3_ support, and CoPc/C, obtained by removing the Al_2_O_3_ template from Al_2_O_3_@C-7 and subsequently loading CoPc molecules (Fig. S3–S6†). N_2_ adsorption–desorption isotherms showed that the specific surface area of the pure carbon substrate derived by removing the Al_2_O_3_ template nearly tripled (Fig. S7†). The characteristic D band at 1334 cm^−1^ and G band at 1597 cm^−1^ of graphene were observed in the Raman spectra of C and Al_2_O_3_@C-3 (Fig. S8†). Raman spectroscopy also confirmed the successful deposition of CoPc on all samples, including CoPc/Al_2_O_3_@C-3, CoPc/Al_2_O_3_ and CoPc/C (Fig. 1f). It should be noted that the Raman signals of graphene in CoPc/Al_2_O_3_@C-3 become less pronounced because they are overlapped with the pyrrole C–C stretch at 1331 cm^−1^ and the benzene C=C stretch at 1596 cm^−1^ in CoPc (Fig. S8†).^41^ Although the Raman characteristic peaks of CoPc in CoPc/Al_2_O_3_@C-3 and CoPc/C are intense, X-ray diffraction spectrometry (XRD) failed to detect any CoPc signals on the two carbonized samples (Fig. S9†), indicating that the deposited CoPc molecules were not in a crystalline form but rather well-dispersed, which is due to the strong π–π interaction between the macrocyclic Pc ring and the underlying graphene.^23,42^ We surmise that the highly dispersed CoPc molecules strongly attached to the few-layer graphene would further induce significant electronic coupling between the CoPc metal center and the Al_2_O_3_ support, which will be scrutinized below using X-ray absorption spectroscopy (XAS) and density functional theory (DFT) calculations.

XPS spectra of CoPc, CoPc/C and CoPc/Al_2_O_3_@C-3 are collected (Fig. S10†). Peaks at 781.0 and 796.4 eV in the Co 2p XPS spectra are attributed to Co 2p_3/2_ and 2p_1/2_ orbitals. Peaks at 399.1 eV, 399.5 eV and 401.0 eV in the N 1s XPS spectra are attributed to pyrrole N, Co–N and N–CO_2_, respectively.^43^ In the Co K-edge X-ray absorption near edge structure (XANES) spectra, all samples of CoPc/Al_2_O_3_@C-3, CoPc/Al_2_O_3_, CoPc/C and pristine CoPc exhibit pre-edge absorption at 7710.5 eV, attributed to the 1s to 3d transition, and an absorption edge at 7716.5 eV, corresponding to the 1s to 4p_*z*_ transition. These features arise from the square-planar Co–N_4_ coordination structure with *D*_4h_ symmetry (Fig. 1g).^44^ Typically, Co–N_*x*_ coordination deviating from the quadrilateral configuration would result in enhanced pre-edge absorption but a reduced peak intensity at the absorption edge. Therefore, it becomes evident that the CoPc molecules supported on Al_2_O_3_@C-3 manifest the most pronounced distortion from the *D*_4h_ symmetry.^34,40,45^ This is likely due to a repulsive interaction between the Co^2+^ center and the Al_2_O_3_ substrate, both of which act as electron acceptors, drawing contributions from the delocalized π orbitals shared by the *P*c ring and the graphene interlayer. Consequently, one might envisage that the macrocyclic ring of CoPc would bend down, pushing the Co^2+^ center away from the substrate plane.

**Fig. 2:**
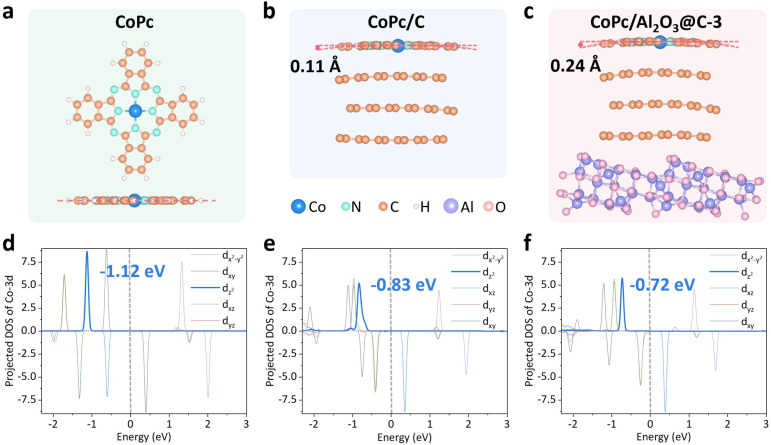
Schematic diagrams of the calculation models and the calculated PDOS of Co 3d orbitals for (a and d) CoPc, (b and e) CoPc/C and (c and f) CoPc/Al_2_O_3_@C, respectively. In (a), a top view (top) and a side view (bottom) of CoPc are shown, while in (b) and (c) only the side views are displayed and the labeled values represent the axial distance from the central Co atom to the peripheral H atoms.

To support the hypothesis above, DFT calculations were performed on three structural models, including pristine CoPc, CoPc on top of tri-layer graphene (CoPc/C), and CoPc laid over graphene-coated Al_2_O_3_. The simulation cell parameters and the corresponding intermolecular and interfacial distances along all three lattice directions of CoPc/C and CoPc/Al_2_O_3_@C-3 are shown in Fig. S11–S14,† which could exclude the influence of periodic structural effects. Indeed, compared to the flat planar configuration of pristine CoPc (Fig. 2a), the same macrocyclic molecule supported on few-layer graphene exhibits a distorted quadrilateral configuration, bending downward with an out-of-plane deflection of about 0.11 Å (Fig. 2b), which is ascribed to the strong π–π stacking between the macrocyclic ring of CoPc and the underlying graphene. The out-of-plane deflection of CoPc increases to 0.24 Å by further introducing the bottom Al_2_O_3_ layer (Fig. 2c), supporting the argument of electrostatic repulsion between the Co^2+^ center and the Al_2_O_3_ substrate. Thus, both the graphene interlayer and the Al_2_O_3_ support interact electronically with CoPc, but through different structural moieties with opposing vectors.

Next, the projected density of states (PDOS) was calculated for Co 3d orbitals in pristine CoPc, CoPc/C and CoPc/Al_2_O_3_@C-3. As anticipated, the energy levels of d_*xz*_, d_*yz*_, d_*z*^2^_, d_*xy*_, and d_*x*^2^−*y*^2^_ from CoPc follow an ascending order on account of the *D*_4h_ symmetry of the Co–N_4_ coordination (Fig. 2d). In the process of eCO_2_R, electrons are typically perceived transferring from the d_*z*^2^_ orbital to the lowest unoccupied molecular orbital (LUMO) of the intermediate adsorbed on the metal center. Thus, the energy level of d_*z*^2^_ of the metal center is paramount to the energetics of the elementary reactions. As shown by the PDOS calculations, the energy level of the Co d_*z*^2^_ orbital in the pristine CoPc is −1.12 eV, which increases to −0.83 eV in CoPc/C and further to −0.72 eV in CoPc/Al_2_O_3_@C-3 (Fig. 2d–f). Therefore, with the increased d_*z*^2^_ energy, CoPc/Al_2_O_3_@C-3 is expected to be more efficient than CoPc and CoPc/C in driving electrolytic CO_2_ reduction. The PDOS analysis was further supported by the CO_2_ uptake normalized by the BET surface area (Fig. S15†) and CO_2_ temperature-programmed desorption (TPD) curves (Fig. S16†). While CoPc/Al_2_O_3_@C-3, CoPc/C, and CoPc exhibit desorption peaks related to weakly adsorbed CO_2_ around 40 °C, only CoPc/Al_2_O_3_@C-3 demonstrates a pronounced desorption peak related to strongly adsorbed CO_2_ at 150 °C, which indicates that Al_2_O_3_@C-3 showed enhanced CO_2_ binding capability.^46^ Moreover, we constructed the model of CoPc loaded on single layer graphene-coated Al_2_O_3_ (CoPc/Al_2_O_3_@C-1), where the out-of-plane deflection of CoPc is 0.17 Å and the energy level of the Co d_*z*^2^_ orbital is −0.76 eV. Both of these values are between the corresponding data for CoPc/C and CoPc/Al_2_O_3_@C-3, demonstrating that CoPc/Al_2_O_3_@C-3 does have the most significant distortion and the highest d_*z*^2^_ energy (Fig. S17†).

### Featured eCO_2_R performance

**Fig. 3:**
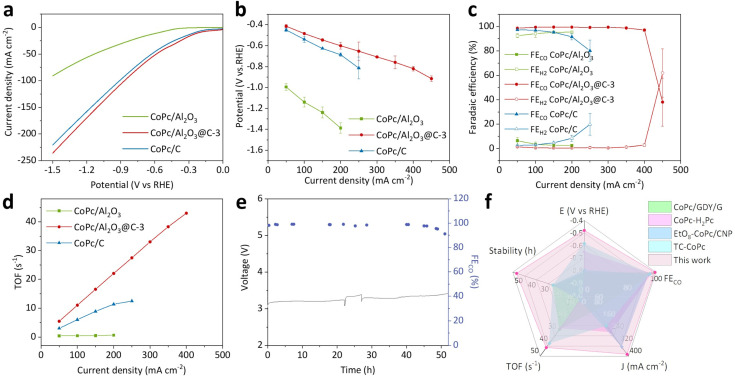
eCO_2_R performance of CoPc/Al_2_O_3_@C-3. (a) LSV curves, (b) galvanostatic *j*–*V* plots, (c) FEs of CO and H_2_ plotted against the current density, and (d) TOFs at varying current densities measured for CoPc/Al_2_O_3_@C-3 in reference to CoPc/Al_2_O_3_ and CoPc/C. (e) Stability test at 100 mA cm^−2^ for CoPc/Al_2_O_3_@C-3 in an MEA (0.1 M KHCO_3_). (f) Radar plot of performance indices with regard to the cathodic potential and operational stability at 100 mA cm^−2^ and maximum FE, TOF and partial current density of CO.

The eCO_2_R performance of CoPc/Al_2_O_3_@C-3, CoPc/Al_2_O_3_ and CoPc/C was evaluated in 1 M KOH electrolyte using a three-electrode flow cell. Both Al_2_O_3_ and Al_2_O_3_@C-3 have no eCO_2_R selectivity with hydrogen being the major product (Fig. S18†). Linear sweep voltammetry (LSV) curves (Fig. 3a) showed that under the same potential, CoPc/Al_2_O_3_@C-3 presented a marginally higher current density compared to CoPc/C, while both significantly outperformed CoPc/Al_2_O_3_. Indeed, in galvanostatic eCO_2_R tests conducted at varying current densities, CoPc/Al_2_O_3_@C-3 exhibited more positive cathodic potentials than CoPc/C, with both catalysts showing significantly higher potentials than CoPc/Al_2_O_3_ (Fig. 3b). More remarkably, CoPc/Al_2_O_3_@C-3 demonstrated a near-unity FE_CO_ across a broad current density range from 50 to 400 mA cm^−2^, achieving a maximum partial current density (*J*_CO_) value of 388 mA cm^−2^ (Fig. 3c). By comparison, CoPc/C can only sustain an operating current density up to 250 mA cm^−2^ before the FE_CO_ drops below 80%, with a maximum *J*_CO_ of 182 mA cm^−2^. For CoPc/Al_2_O_3_, H_2_ was the dominant reduction product, accounting for over 90% of the faradaic efficiency throughout the test. This result indicates that CoPc failed to effectively catalyze eCO_2_R on the insulating Al_2_O_3_ substrate, highlighting the critical role of the conductive support in facilitating the redox reaction.

For all three samples, the TOFs for eCO_2_R were calculated based on the Co content (Table S1†), as quantified by inductively coupled plasma-atomic emission spectrometry (ICP-AES). CoPc/Al_2_O_3_@C-3 demonstrated a maximum TOF of 43 s^−1^, significantly higher than those of CoPc/C (12 s^−1^) and CoPc/Al_2_O_3_ (0.6 s^−1^) (Fig. 3d). The exceptional TOF of CoPc/Al_2_O_3_@C-3 aligns well with the aforementioned high intrinsic activity originating from metal center activation. Furthermore, at 100 mA cm^−2^, CoPc/Al_2_O_3_@C-3 was able to sustain a long-term operation for over 52 hours in a zero-gap MEA (0.1 M KHCO_3_) electrolyzer before the FE_CO_ dropped below 90% (Fig. 3e). After the prolonged electrolysis, no morphological change was observed for the recovered CoPc/Al_2_O_3_@C-3 catalyst, retaining its original nanosheet structure with a distinct graphene coating (Fig. S19†). Raman still exhibits significant CoPc characteristic peaks after electrolysis (Fig. S20†), and the crystal structure of γ-phase Al_2_O_3_ can also be observed in the XRD pattern (Fig. S21†), indicating the moderate electrochemical stability of CoPc/Al_2_O_3_@C-3. Based on the comprehensive evaluation of eCO_2_R performance metrics, CoPc/Al_2_O_3_@C-3 stands out as one of the best-performing CoPc-based catalysts reported for CO production (Fig. 3f and Table S2†).^14,21,47,48^

### Modulating the layer number of the interlayer graphene

**Fig. 4:**
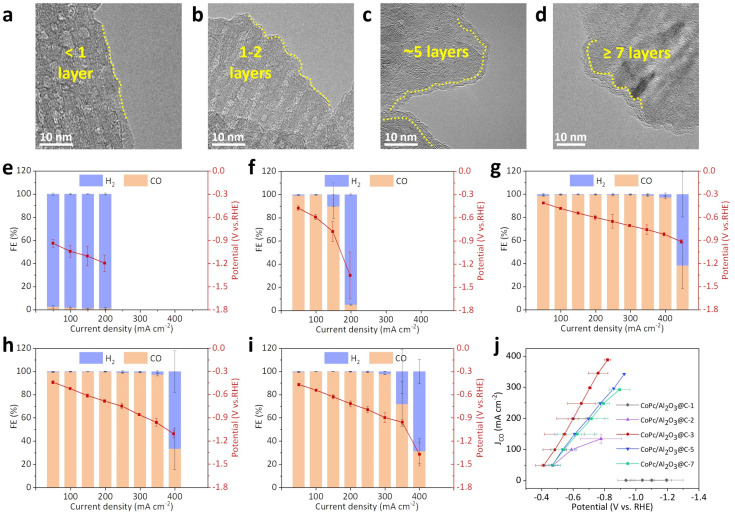
Characterization and eCO_2_R performance of CoPc/Al_2_O_3_@C-*x*. TEM images of (a) CoPc/Al_2_O_3_@C-1, (b) CoPc/Al_2_O_3_@C-2, (c) CoPc/Al_2_O_3_@C-5 and (d) CoPc/Al_2_O_3_@C-7. FEs of CO and H_2_ at varying current densities with the corresponding cathodic potentials for (e) CoPc/Al_2_O_3_@C-1, (f) CoPc/Al_2_O_3_@C-2, (g) CoPc/Al_2_O_3_@C-3, (h) CoPc/Al_2_O_3_@C-5 and (i) CoPc/Al_2_O_3_@C-7. (j) Plotting of the CO partial current densities against the cathodic potentials for all CoPc/Al_2_O_3_@C-*x* samples.

The impact of the interlayer graphene thickness on the eCO_2_R performance was investigated by varying the *n*-hexane feed during CVD to systemically modulate the graphene layer numbers, which were confirmed by HR-TEM images (Fig. 4a–d), thermogravimetric analysis (TGA, Fig. S22†), and ICP-quantified Al contents (Table S3†). Therefore, the *x* value in CoPc/Al_2_O_3_@C-*x* was estimated to be 1, 2, 3, 5, and 7, representing the rounded number of graphene layers. In general, Raman spectra of Al_2_O_3_@C-*x* indicated that the D/G band intensity ratios are within the range of 1.20 to 1.31, suggesting that Al_2_O_3_@C-*x* shows similar graphitization degrees (Fig. S23†). The electric conductivity of Al_2_O_3_@C-*x*, as measured by the four-point probe method, increased with higher *x* values (Fig. S24†). Note that the conductivity difference between Al_2_O_3_@C-1 and Al_2_O_3_ was negligible, implying the presence of a discontinuous graphene layer (Fig. 4a). When *x* increased to 7 (Fig. 4d), the conductivity of Al_2_O_3_@C-7 approached that of pure carbon obtained by removing the Al_2_O_3_ template. Similar to the conductivity measurements, the LSV current density of CoPc/Al_2_O_3_@C-1 was comparable to that of CoPc/Al_2_O_3_ (Fig. S25†). As *x* increased from 2 to 7, the LSV current densities improved significantly, but the trend followed a volcano shape, with CoPc/Al_2_O_3_@C-3 exhibiting the highest current density. This observation suggests that a moderate graphene thickness is optimal for maximizing the eCO_2_R kinetics on the cathode.

Galvanostatic eCO_2_R tests at varying operational current densities showed that the performance of CoPc/Al_2_O_3_@C-1 closely resembled that of CoPc/Al_2_O_3_, with minimal CO generation across the entire tested current range (50–200 mA cm^−2^, Fig. 4e). This, again, can be attributed to the incomplete graphene coverage on the Al_2_O_3_ substrate that undermines the electronic conductivity. CoPc/Al_2_O_3_@C-2 exhibited a high FE_CO_ of 99% at 50 and 100 mA cm^−2^ (Fig. 4f), indicating that the catalyzing effect of CoPc began to take effect. However, H_2_ production still dominated the faradaic process on the cathode when the current density increased to 200 mA cm^−2^. As aforementioned, CoPc/Al_2_O_3_@C-3 sustained a high FE_CO_ of >97% up to 400 mA cm^−2^, showcasing the best performance among all tested samples (Fig. 4g). Further increasing *x* led to a decline in the catalytic performance of CoPc/Al_2_O_3_@C-5 and CoPc/Al_2_O_3_@C-7 (Fig. 4h and i), trending toward the performance of CoPc/C as observed earlier. Notably, CoPc/Al_2_O_3_@C-3 not only achieved the highest *J*_CO_ values among all samples with varying graphene thicknesses but also required a significantly lower cathodic potential to achieve the same *J*_CO_ (Fig. 4j). These comparative studies signify the delicate balance between electric conductivity and catalyst–support interactions necessary to sustain high eCO_2_R activity in CoPc/Al_2_O_3_@C-*x* catalysts.

**Fig. 5:**
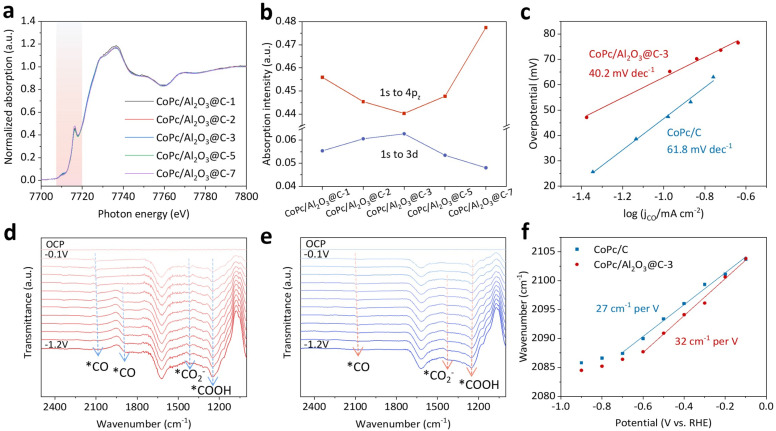
Mechanistic investigation into the graphene-modulated electronic interaction between CoPc and Al_2_O_3_. (a) Co K-edge XANES spectra and (b) trends of pre-edge and edge absorption peaks at 7710.5 and 7716.5 eV, respectively, for CoPc/Al_2_O_3_@C-*x*. (c) Tafel slopes measured for CoPc/Al_2_O_3_@C-3 and CoPc/C. (d) *In situ* ATR-SEIRAS spectra taken on CoPc/Al_2_O_3_@C-3 and (e) CoPc/C by ramping down the cathodic potential from OCP to −1.2 V. (f) Comparison of the Stark shift of *CO near 2100 cm^−1^ for CoPc/Al_2_O_3_@C-3 and CoPc/C.

XANES spectra of CoPc/Al_2_O_3_@C-*x* with varying graphene thickness were analyzed to scrutinize the graphene-mediated catalyst–support interaction (Fig. 5a). As previously discussed, the intensity drop of the edge absorption peak at 7716.5 eV, concurrent with the increasing pre-edge absorption at 7710.5 eV, serves as an indicator of the structural distortion in the quadrilateral Co–N_4_ coordination of CoPc. By plotting the intensity of the edge absorption at 7716.5 eV against the number of graphene layers (Fig. 5b), an inverted volcano relationship becomes evident, with CoPc/Al_2_O_3_@C-3 exhibiting the lowest peak intensity. This indicates the most pronounced deviation from the square-planar *D*_4h_ symmetry of CoPc. Conversely, the pre-edge absorption at 7710.5 eV follows the opposite trend. These findings strongly suggest that an excessively thick graphene interlayer, despite the enhanced π–π stacking and improved electronic conductivity, might shield the electronic interaction between CoPc and Al_2_O_3_, thereby damping their mutual repulsion. This shielding effect likely reduces the catalytic activity of the metal center, highlighting the importance of optimizing graphene thickness to balance these competing factors.

### Mechanistic investigation into the eCO_2_R cascade

Previous DFT calculations have shown that the trilateral interaction among CoPc, graphene, and Al_2_O_3_ bends down the macrocyclic molecule through π–π stacking and electronic repulsion, raising the energy level of d_*z*^2^_ to enable a higher catalytic activity of the metal center. This, in theory, should alter the energetics of the elementary reactions and thereby the overall reaction kinetics. To further understand the observed performance differences, we measured the Tafel slopes of the eCO_2_R reactions on CoPc/C and CoPc/Al_2_O_3_@C-3, aiming to identify the RDSs. The Tafel slope for CoPc/C was found to be 61.8 mV dec^−1^ (Fig. 5c), pointing to a slow chemical reaction initiated by an electron transfer step as the RDS (eqn (1) and(2)).^19^

1 *CO_2_ + e^−^ → *CO_2_^−^

2 *CO_2_^−^ + H^+^ → *COOH

By comparison, the Tafel slope for CoPc/Al_2_O_3_@C-3 was 40.2 mV dec^−1^, suggesting that the RDS is a fast proton-coupled electron transfer (PCET) step, corresponding to either eqn (3) or(4).

3 *CO_2_ + H^+^ + e^−^ → *COOH

4 *COOH + H^+^ + e^−^ → *CO + H_2_O

Therefore, the elevated Co d_*z*^2^_ orbital in CoPc/Al_2_O_3_@C-3 not only facilitates CO_2_ activation but also reduces the energy barriers of elementary reactions, greatly expediting the eCO_2_R kinetics.

To trace the evolution of intermediates involved in these reaction pathways, *in situ* attenuated total reflectance surface-enhanced infrared absorption spectroscopy (ATR-SEIRAS) was conducted (Fig. 5d and e). Owing to the largely shared reaction pathway and intermediates, both CoPc/Al_2_O_3_@C-3 and CoPc/C exhibited similar IR signatures in the real-time spectra acquired while ramping down the applied potential from the open-circuit potential (OCP) to −1.2 V. However, a close inspection can still reveal subtle differences in details. For CoPc/Al_2_O_3_@C-3, the early observation of *CO_2_^−^ at high potentials confirms that CO_2_ activation is facilitated. Additionally, the peak corresponding to *COOH at 1248 cm^−1^, intensifying with decreasing potential, indicates that the second PCET step (eqn (4)) is likely the RDS.^40^ Combining these IR observations with the Tafel slope measurements, the eCO_2_R reaction cascade on CoPc/Al_2_O_3_@C-3 can be deduced as eqn (1),(2) and (4), with the last PCET step being rate-determining. In contrast, on CoPc/C the second chemical reaction step is more sluggish, significantly reducing the eCO_2_R kinetics and selectivity.

Another notable difference between the two sets of IR spectra lies in the emerging *CO peak at 1885 cm^−1^ observed for CoPc/Al_2_O_3_@C-3 (Fig. 5d), indicating the emergence of a different vibration mode (likely related to *=C=O) at more negative potentials.^45^ Concurrently, the peak at 2100 cm^−1^, corresponding to *–C≡O, decreased in intensity with increasing bias. This transition in the *CO vibration mode reflects the dynamic behavior of intermediates, further corroborating the high catalytic activity of CoPc/Al_2_O_3_@C-3 in converting CO_2_ to CO. Additionally, a bias-dependent frequency shift was observed for *CO at 2100 cm^−1^ on both CoPc/Al_2_O_3_@C-3 and CoPc/C, attributed to the combined effects of Fano line shape variation (related to coverage) and Stark tuning (related to the local electric field) (Fig. 5f).^49^ The Stark tuning rate of the *CO band on CoPc/Al_2_O_3_@C-3 was 32 cm^−1^ V^−1^, which is higher than that of CoPc/C (27 cm^−1^ V^−1^). This indicates that the *CO adsorption on CoPc/Al_2_O_3_@C-3 is more sensitive to the electric field while less affected by competition from *H adsorption.

**Fig. 6:**
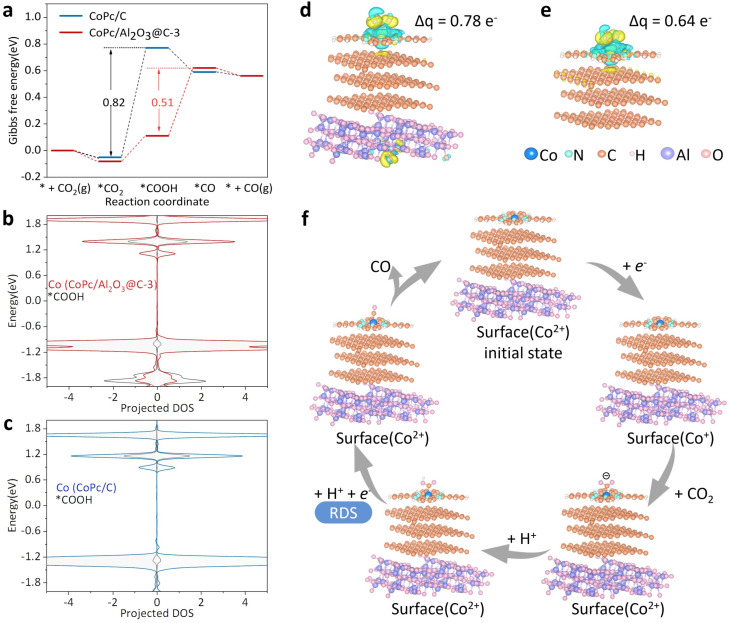
DFT calculations on the intermediates and reaction energetics of eCO_2_R. (a) Gibbs free energy diagrams of intermediates on CoPc/Al_2_O_3_@C-3 and CoPc/C along the eCO_2_R reaction coordinate. (b and c) PDOS of *COOH adsorbed on CoPc/Al_2_O_3_@C-3 and CoPc/C, respectively. (d and e) 3D charge density difference plots for CoPc/Al_2_O_3_@C-3 and CoPc/C, respectively, (Δ*q* represents the differential charge density from Bader charge analysis of adsorbed *COOH). (f) The proposed reaction pathway on CoPc/Al_2_O_3_@C-3 with a shifted RDS (from that of CoPc/C).

DFT calculations were performed to elucidate the structure and energetics of intermediates involved in eCO_2_R for CO production. Fig. 6a displays the Gibbs free energy and formation energy profiles of the intermediates along the reaction coordinate for CoPc/Al_2_O_3_@C-3 and CoPc/C. On CoPc/C, *COOH has a high free energy, making this step (eqn (3)) the RDS, with an energy barrier of 0.82 eV. By contrast, the free energy of *COOH on CoPc/Al_2_O_3_@C-3 is much lower, shifting the RDS to eqn (4) with a formation energy of 0.51 eV. This result aligns well with the IR observation of a strong *COOH signal on CoPc/Al_2_O_3_@C-3 and further agrees with the Tafel slope measurements for RDS determination. Thus, the introduction of the Al_2_O_3_ support facilitates CO_2_ activation, shifts the RDS, and accelerates the reaction kinetics.

As shown in Fig. 6a, the most prominent difference in the free energy of intermediates between CoPc/Al_2_O_3_@C-3 and CoPc/C is from *COOH, which shifts the paradigm of the reaction energetics. To further investigate *COOH binding, PDOS analysis was conducted (Fig. 6b, c, S26 and S27†). For *COOH adsorbed on CoPc/Al_2_O_3_@C-3, the resonance between its bonding and antibonding orbitals is significantly stronger than that on CoPc/C, indicating enhanced electronic orbital hybridization. Additionally, the differential charge density and Bader charge analysis of *COOH adsorbed on CoPc/Al_2_O_3_@C-3 and CoPc/C (Fig. 6d and e) revealed that the Bader charge of *COOH on CoPc/Al_2_O_3_@C-3 is 0.78e^−^, significantly higher than that on CoPc/C (0.64e^−^). This suggests that *COOH receives more charge from CoPc/Al_2_O_3_@C-3, further supporting the stronger electron-donating capability of the elevated d_*z*^2^_ orbital. This is corroborated by the smaller O–C=O bond angle and shorter Co–C bond length observed for *COOH on CoPc/Al_2_O_3_@C-3 (Table S4†).

Finally, based on the comprehensive experimental and theoretical evidence presented above, the eCO_2_R reaction cascade on both CoPc/Al_2_O_3_@C-3 and CoPc/C can be delineated, sharing the same pathway but quite different energetics (Fig. 6f). The process begins with the metal center of CoPc accepting one electron, converting to Co^+^, which then relays the electron to the adsorbed CO_2_ molecule, forming the *CO_2_^−^ intermediate observed in the IR spectra. This initial step is greatly facilitated on CoPc/Al_2_O_3_@C-3 through the elevated d_*z*^2^_ orbital of Co due to symmetry breaking. Next, *CO_2_^−^ accepts a proton to form *COOH, which is more stable on CoPc/Al_2_O_3_@C-3 and produces a strong IR signal. Subsequently, through a PCET process *COOH is converted to *CO. The substantial free energy difference of *COOH between CoPc/C and CoPc/Al_2_O_3_@C-3 shifts the RDS from the *COOH formation step for CoPc/C to the *CO formation step for CoPc/Al_2_O_3_@C-3. Finally, *CO is released from both catalysts through an exothermic process, completing the reaction cycle.

## Conclusions

In this study, aiming to modulate the catalyst–support interaction, graphene-skinned Al_2_O_3_ was employed to load CoPc molecules for driving electrochemical CO_2_ reduction. The strong π–π stacking between the macrocyclic Pc ring and the graphene interlayer, coupled with the electronic repulsion between the divalent metal center and the underlying Al_2_O_3_ substrate, induces a downward bending of the CoPc molecule, deviating from its square-planar configuration with a distorted *D*_4h_ symmetry. This structural change realigns the Co 3d orbitals, particularly raising the energy level of d_*z*^2^_. Consequently, the deformed CoPc molecule, with its activated metal center, enhances CO_2_ activation, lowers the free energy of *COOH, shifts the rate-determining step, and thereby accelerates the overall eCO_2_R kinetics. These effects were well supported by synchrotron spectroscopy, Tafel measurements, *in situ* ATR-SEIRAS and DFT calculations. The best catalyst, CoPc/Al_2_O_3_@C-3, with an optimal graphene thickness, achieved a near-unity FE_CO_ across a wide current range, an exceptional TOF of 43 s^−1^, a low overpotential of 0.7 V at 400 mA cm^−2^, and a prolonged stability for CO production in an MEA. This study, by leveraging the vectorial interactions between molecular moieties and the substrate to reshape the macrocyclic structure and realign the orbital energies of CoPc, provides new insights into the engineering of catalyst–support interactions for enhanced eCO_2_R activities. These findings can potentially be extended to other similar composite systems, opening new avenues for the design of efficient electrocatalysts.

## Data availability

The data supporting this article have been included as part of the ESI.†

## Author contributions

Qianqian Bai: data curation; formal analysis; methodology; visualization; investigation; writing – original draft. Bingyun Ma: data curation; formal analysis. Le Wei: data curation; formal analysis. Mutian Ma: data curation; formal analysis; investigation. Zhangyi Zheng: visualization. Wei Hua: data curation. Zhenyang Jiao: formal analysis. Min Wang: data curation. Huihong Yuan: data curation. Zhihe Wei: data curation. Tao Cheng: software. Xiaoxing Ke: data curation. Jun Zhong: data curation; formal analysis. Fenglei Lyu: conceptualization; formal analysis; funding acquisition; writing – review & editing. Zhao Deng: conceptualization; formal analysis; funding acquisition; supervision; writing – review & editing. Yang Peng: conceptualization; formal analysis; funding acquisition; supervision; writing – review & editing.

## Conflicts of interest

There are no conflicts to declare.

## Supplementary Material

SC-OLF-D5SC02616D-s001
