# Supplementary material for: Engineering catalyst–support interactions in cobalt phthalocyanine for enhanced electrocatalytic CO2 reduction: the role of graphene-skinned Al2O3
Source: Chem Sci. 2025 May 22;16(25):11587–97. doi: 10.1039/d5sc02616d (PMC12123375; doi:10.1039/d5sc02616d)
Supplement: SC-016-D5SC02616D-s001 [file SC-016-D5SC02616D-s001.pdf]

Supplementary Information (SI) for Chemical Science.
This journal is © The Royal Society of Chemistry 2025

Supporting Information

Engineering Catalyst-Support Interactions in Cobalt Phthalocyanine for Enhanced Electrocatalytic CO_2 Reduction: The Role of Graphene-Skinned Al_2O_3

Qianqian Bai, ‡[a] Bingyun Ma, ‡[b] Le Wei,[c] Mutian Ma,[a] Zhangyi Zheng,[a] Wei Hua,[a] Zhenyang Jiao,[a] Min Wang,[a] Huihong Yuan,[a] Zhihe Wei,[a] Tao Cheng,[b] Xiaoxing Ke,[c] Jun Zhong,[b] Fenglei Lyu,*[a] Zhao Deng*[a] and Yang Peng*[a]

a. Soochow Institute of Energy and Material Innovations, College of Energy, Jiangsu Key Laboratory for Advanced Negative Carbon Technologies, Soochow University, Suzhou 215006, China

b. Institute of Functional Nano & Soft Materials (FUNSOM), Soochow University, Suzhou 215123, China

c. Beijing Key Laboratory of Microstructure and Properties of Solids, College of Materials Science and Engineering, Beijing University of Technology, Beijing 100124, China

* E-mail: fllv@suda.edu.cn, zdeng@suda.edu.cn, ypeng@suda.edu.cn
‡ These authors contributed equally to this work.

Materials and Methods

Materials

Aluminium chloride hexahydrate ($AlCl_3·6H_2O$, 99%) were purchased from Alfa Aesar, Thermo Fisher Scientific Co., Ltd. Hexamethylenetetramine ($C_6H_{12}N_4$, HMT, 99%), potassium hydroxide (KOH, ≥95%) and ethanol (C_2H_6O, ≥99.7%) were purchased from Sinopharm Chemical Reagent Co., Ltd. N-hexane (C_6H_{14}, 99.9%) was purchased from Saen Chemical Technology (Shanghai) Co., Ltd. Cobalt phthalocyanine (CoPc, 99%), was purchased from Shanghai Aladdin Bio-Chem Technology Co., Ltd. N, N-dimethylformamide (DMF, 99.5%) was purchased from Shanghai Macklin Biochemical Technology Co., Ltd. All reagents were used as received without further purification. Deionized (DI) water was purified with a Sartorius ultrapure water system.

Synthesis of AlO(OH)

Initially, 1.207 g of $AlCl_3·6H_2O$ and 0.84 g of HMT were completely dissolved in a mixed solvent consisting of 30 mL deionized water and 30 mL DMF. Then the solution was transferred into a 100 mL Teflon-lined stainless steel autoclave and maintained at 150℃ for 8 h to form AlO(OH) microplates. After naturally cooling to room temperature, the product was washed with deionized water and ethanol, centrifuged, and then dried in a vacuum oven at 80℃ overnight.

Synthesis of CoPc/Al_2O_3/C-x (x=1, 2, 3, 5, 7)

To synthesize the graphene-skinned Al_2O_3 support, 300 mg of the previously prepared AlO(OH) powder was placed into a tube furnace purged with y (y = 2, 2.5, 3, 4.5, 6) mL of the vaporized n-hexane. The quartz tube was heated to 900℃ and maintained at this temperature for 2 h to fully convert AlO(OH) into Al_2O_3 and carbonize the n-hexane into graphene. The obtained products are denoted as Al_2O_3@C-x, where x represents the rounded number of graphene layers coating the Al_2O_3.

Next, CoPc was dissolved in DMF at a concentration of 0.5 mg mL^{-1}. Then, 50 mg of the Al_2O_3@C-x powder was dispersed in 20 ml of DMF and sonicated for at least

30 min. The above CoPc/DMF solution was then added to the Al_2O_3@C-x/DMF dispersion under continuous sonication, with a CoPc mass ratio of 5%, and then stirred for 24 h at room temperature to obtain CoPc/Al_2O_3@C-x. Afterwards, the product was collected and washed with DMF and ethanol by centrifugation, and dried thoroughly in vacuum oven.

Synthesis of CoPc/Al_2O_3

To synthesize the bare Al_2O_3 support, 300 mg of AlO(OH) powder was placed into a tube furnace and heated to 900°C. After annealing for 2 h, AlO(OH) was completely converted to Al_2O_3. CoPc was then loaded onto the Al_2O_3 support following the same method as described for CoPc/Al_2O_3@C-x.

Synthesis of CoPc/C

To synthesize the pure carbon support for control studies, the Al_2O_3 substrate was removed from Al_2O_3@C-7 by immersing in 10% HF for 36 h. The carbonaceous powder was then filtered and thoroughly washed with deionized water and ethanol until neutral. Afterwards, the product was dried completely in vacuum overn. CoPc was then loaded onto the carbon support following the same method as described for CoPc/Al_2O_3@C-x.

Characterization

SEM images were obtained from a Hitachi SU8010 scanning electron microscope with an accelerating voltage of 10 kV. TEM images were collected on an FEI TECNAI G20 field-emission transmission electron microscope with an accelerating voltage of 200 kV. The amounts of Co and Al elements were quantified by an OPTIMA 8000 Inductively Coupled Plasma-Atomic Emission Spectrometer (ICP-AES, PerkinElmer). The crystal structure analysis was conducted by a D8 Advance X-ray diffractometer (XRD, Bruker) using Cu-Ka radiation. Raman spectra were collected on an spectro-electrochemical system (Dowell iHR550) with a CCD from HORIBA. In situ ATR-SEIRAS measurements were conducted using a Fourier transform infrared spectroscopy (Nicolet iS50 FT-IR, Thermo Scientific). XPS data were acquired on an Escalab 250Xi X-ray photoelectron spectrometer (Thermo Fisher) with Al Ka (1486.6 eV) X-rays as the excitation source. X-ray Absorption Near Edge Structure (XANES)

and Extended X-ray Absorption Fine Structure (EXAFS) were analyzed on Beamline 11B at the Shanghai Synchrotron Radiation Facility. N_2 adsorption-desorption isothers, pore-size distribution and CO_2 uptake capacities were measured on an ASAP 2460 automatic BET specific surface (area) analyzer (Micromeritics). Electric conductivity of the powder samples were measured by an ST2263 digital four-probe tester (Suzhou Jingge Electronic) by pressing the powders into pellets. TGA curves were obtained from the TG/DTA7300 Thermogravimetric Analyzer (SII NanoTechnology).

Electrochemical measurements

To prepare the catalyst ink, 10 mg of the CoPc/Al_2O_3@C-x catalyst was dispersed into a solution containing 1 mL of ethanol and 0.1 mL of 5 wt% Nafion under sonication, forming a uniform slurry. Then, the catalyst ink was drop-casted on the gas diffusion layer (GDL, YLS-30T) with a mass loading of 1 mg cm^{-2} and completely dried at room temperature. Electrochemical CO_2 reduction was performed by using the CHI660E electrochemical workstation and carried out in a flow cell, consisting of a nickel foam anode, a gas diffusion electrode (GDE) and an anion exchange membrane (Fumasep FAB-PK-130). Ag/AgCl electrode was used as the reference electrode. 1 M KOH electrolyte was cycled through the cathodic and anodic chambers at a flow rate of 20 ml min^{-1} through a peristaltic pump. During the measurements, high purity CO_2 was bubbled into the gas chamber at a constant flow rate of 30 ml min^{-1} controlled by a digital mass flow controller. The gas products (CO and H_2) were quantitatively analyzed using gas chromatography equipped with both the flame ionization and thermal conductivity detectors (Agilent 7890B). All potentials were converted to RHE according to the equation E(vs. RHE) = E(vs. Ag/AgCl) + 0.198 + 0.059pH + iR, where R represents the resistance between the working electrode and reference electrode, determined using the i-interrupt method with an 85% ohmic resistance (iR) correction applied.

Stability tests were carried out in a membrane electrode assembly with an area of 5 cm^2. Ti mesh loaded with 1 mg cm^{-2} of IrO_2 was used as catalyst at the anode and 0.1 M $KHCO_3$ was passed through as the electrolyte. The cathode was fluxed with

humidified CO_2 at a flow rate of 60 ml min^{-1}, and the above catalyst ink was sprayed on the GDL at a loading of 1 mg cm^{-2} as the cathode GDE. A sustainion membrane (Dioxide Materials, X37-50) was used as an anion exchange membrane between the cathode and anode.

The turnover frequency (TOF) of different samples was calculated respectively according to the Co content with the following formula:

$$TOF\ (s^{-1}) = \frac{j/nF}{m_{cat} \times w/M_{Co}}$$

where j is the partial current (A) of the target product CO, n is the number of electron transfers required to generate 1 mol target product (n is 2 for CO), F is the Faraday's constant (96485 C mol^{-1}), m_{cat} is the mass of the catalyst (g), w is the weight percentage of Co in the catalyst (wt%), and M_{Co} is the atomic weight of Co (58.93 g mol^{-1}).

In situ ATR-SEIRAS measurements

In situ attenuated total reflection-surface enhanced infrared absorption spectroscopy (ATR-SEIRAS) measurements were performed on an FT-IR spectrometer equipped with a mercury cadmium telluride (MCT) detector, using a three-electrode H-cell. A 150 nm gold film was first deposited onto a single-crystal silicon prism via electron-beam physical vapor deposition (PVD) at 250 °C to enhance IR signal intensity and substrate conductivity. The catalyst ink was then drop-coated onto the gold-modified prism at a loading of 1 mg cm^{-2}, and the working electrode was prepared after drying at room temperature. The ATR-SEIRAS cell consisted of the working electrode, a platinum wire counter electrode, and a standard Ag/AgCl reference electrode, with 0.1 M $KHCO_3$ as the electrolyte. Prior to testing, high-purity CO_2 was bubbled into the cathodic electrolyte at 40 mL min^{-1} for at least 15 min to ensure saturation. During electrochemical testing, controlled by a CHI660E electrochemical workstation, a continuous CO_2 flow (40 mL min^{-1}) and an N_2 purge (20 mL min^{-1}) were maintained. Open-circuit potential (OCP) IR spectra were recorded for background subtraction, and chronoamperometric tests were conducted from −0.1 V to −1.2 V (vs. Ag/AgCl) with a step interval of 0.1 V and a scan

rate of 0.05 V s^{-1}. IR spectra were collected every 50 s, with each spectrum averaged over 32 scans at a resolution of 4 cm^{-1}. Additional instrument parameters included a 120 μm aperture and a mirror velocity of 1.8.

Calculation Methods

All calculations in this study were conducted using the Vienna Ab initio Simulation Package (VASP) version 5.4.4 with the framework of spin-polarized density functional theory (DFT). [1, 2] The Perdew-Burke-Ernzerhof (PBE) algorithm within the Generalized Gradient Approximation (GGA) framework was utilized for exchange-correlation interaction. The DFT-D3 method was used to improve the handling of long-range van der Waals interactions. [3, 4] Based on XRD results, a six-layer (2×2) supercell slab with Al_2O_3 (110) was selected for calculations. An energy cutoff of 400 eV and a Monkhorst-Pack grid with a k-point mesh of 1×1×1 and 3×3×1 were used for geometry optimizations and electronic structure property calculations, respectively. The energy convergence criterion during electronic self-consistency iterations was set to 1×10^{-6} eV, and all ionic relaxation loops were terminated when forces fell below 0.01 eV $Å^{-1}$. To avoid interlayer interaction, we set the vacuum space in the z direction to 30 Å. All atoms were permitted to relax during the optimization of the structure. Periodic calculations were carried out to analyze the charge populations using Bader charge analysis. [5]

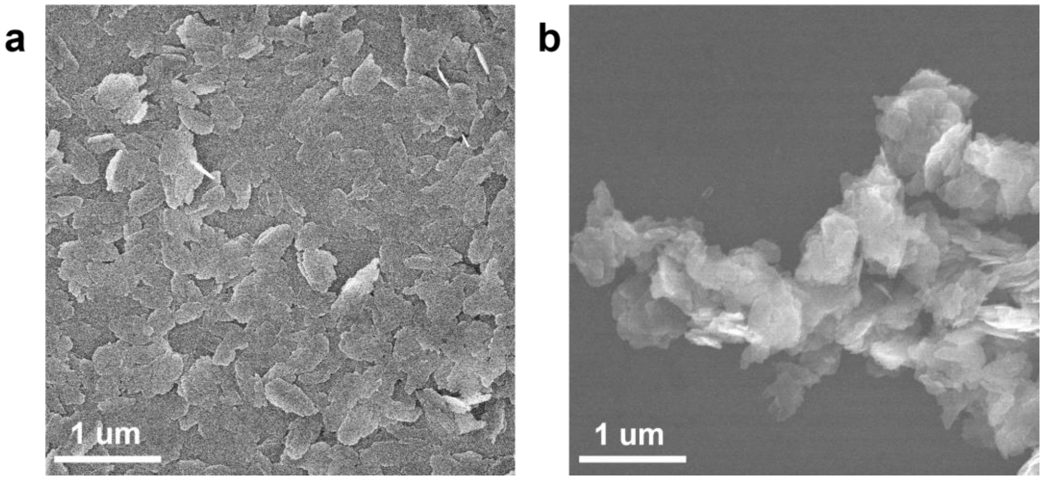

Figure S1. SEM images of (a) AlO(OH) and (b) Al_2O_3@C-3.

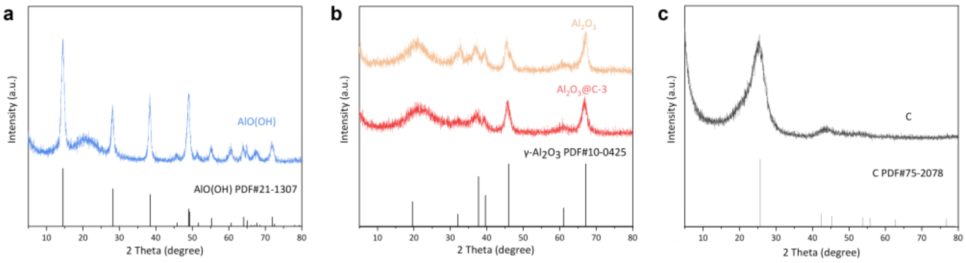

Figure S2. XRD patterns of (a) AlO(OH), (b) Al_2O_3, Al_2O_3@C-3 and (c) C.

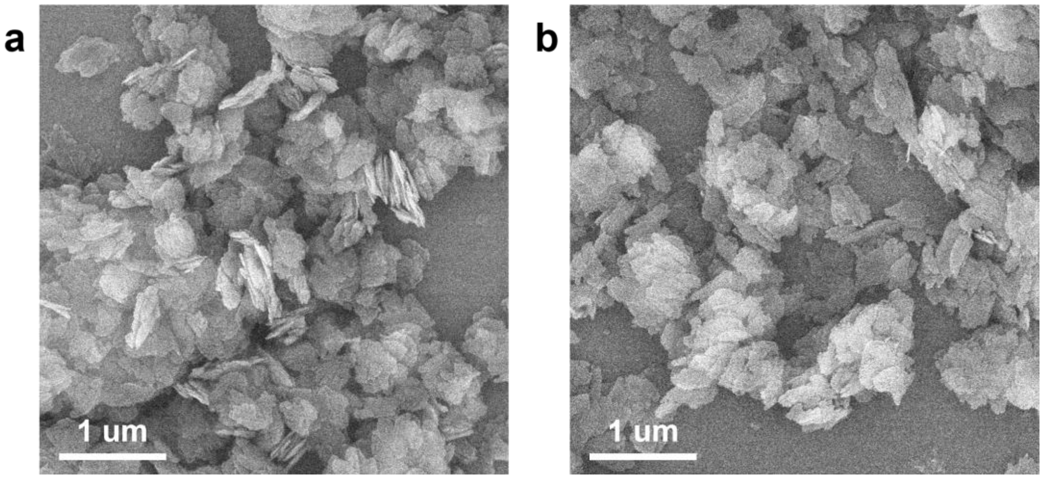

Figure S3. SEM images of (a) Al_2O_3 and (b) CoPc/Al_2O_3.

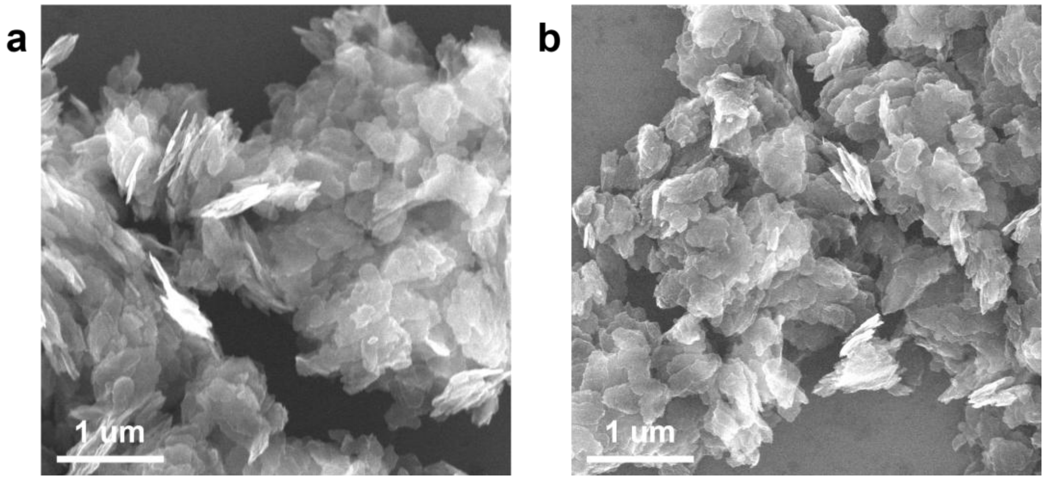

Figure S4. SEM images of (a) C and (b) CoPc/C.

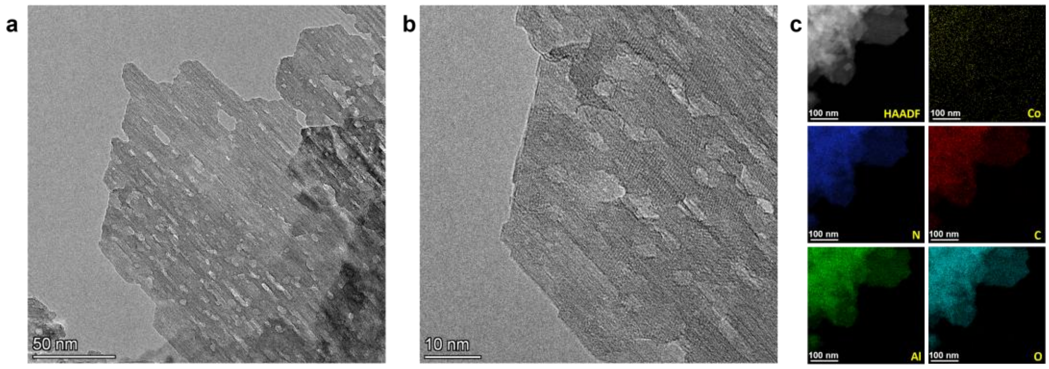

Figure S5. (a) TEM image, (b) HRTEM image and (c) the corresponding EDS elemental mapping images of $CoPc/Al_2O_3$.

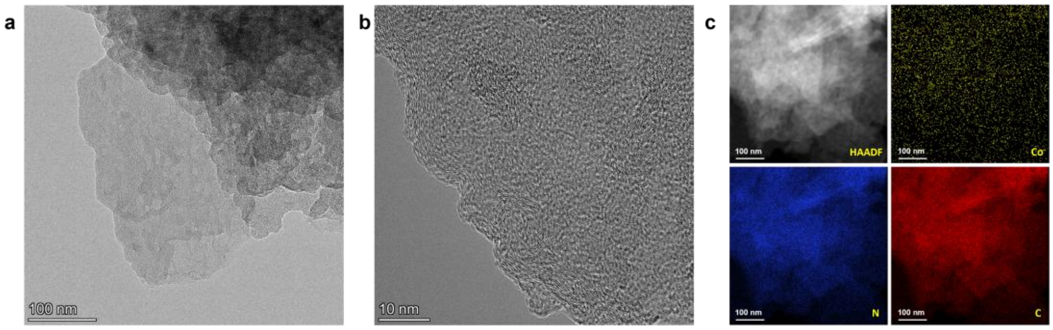

Figure S6. (a) TEM image, (b) HRTEM image and (c) the corresponding EDS elemental mapping images of CoPc/C.

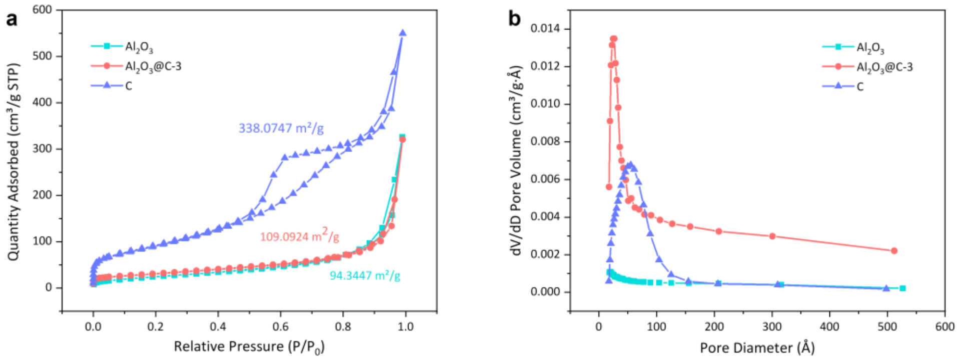

Figure S7. (a) N_2 adsorption-desorption isotherms and (b) pore-size distribution analyses of Al_2O_3, Al_2O_3@C-3 and C.

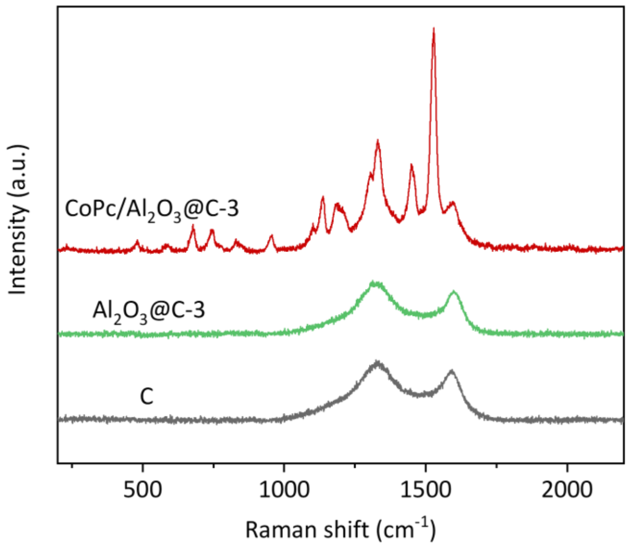

Figure S8. Raman spectra of CoPc/Al_2O_3@C-3, Al_2O_3@C-3 and C.

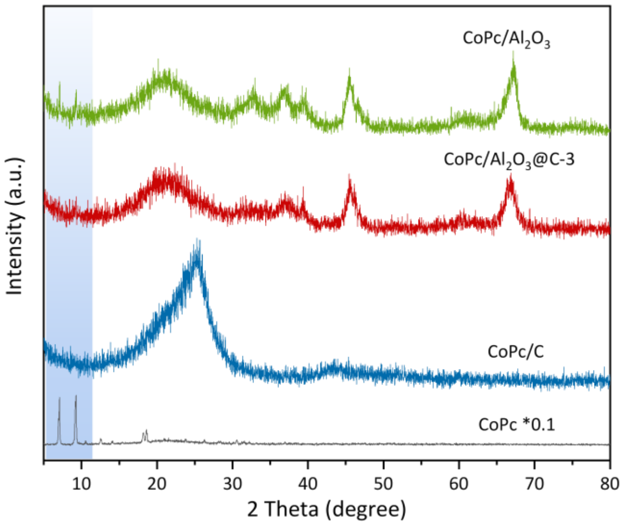

Figure S9. XRD patterns of CoPc/Al_2O_3, CoPc/Al_2O_3@C-3, CoPc/C and CoPc.

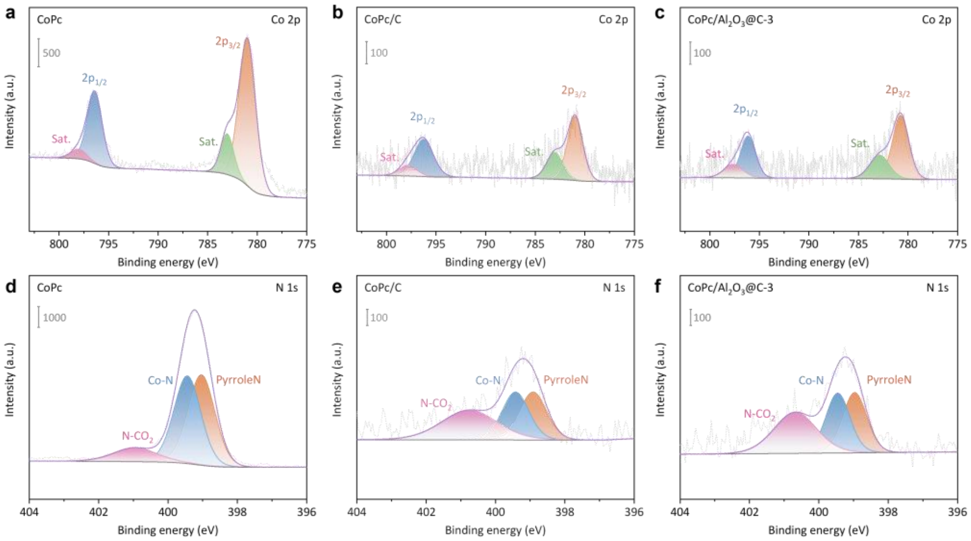

Figure S10. Co 2p and N 1s XPS spectra of (a, d) CoPc, (b, e) CoPc/C and (c, f) $CoPc/Al_2O_3$@C-3.

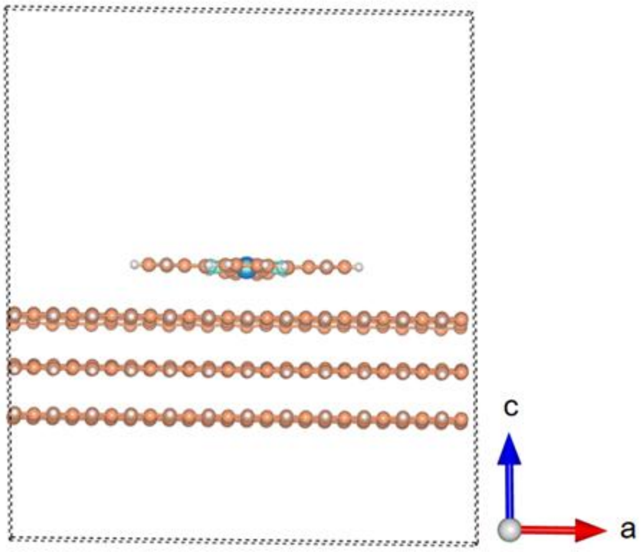

Figure S11. Structural model corresponding to CoPc/C.

The simulation cell parameters of CoPc/C are clarified as follows:

a = 31.11 Å	α = 88.78°

b = 19.49 Å	β = 91.20°
c = 35.46 Å	γ = 90.07°
Volume = 21481.78 Å^3	

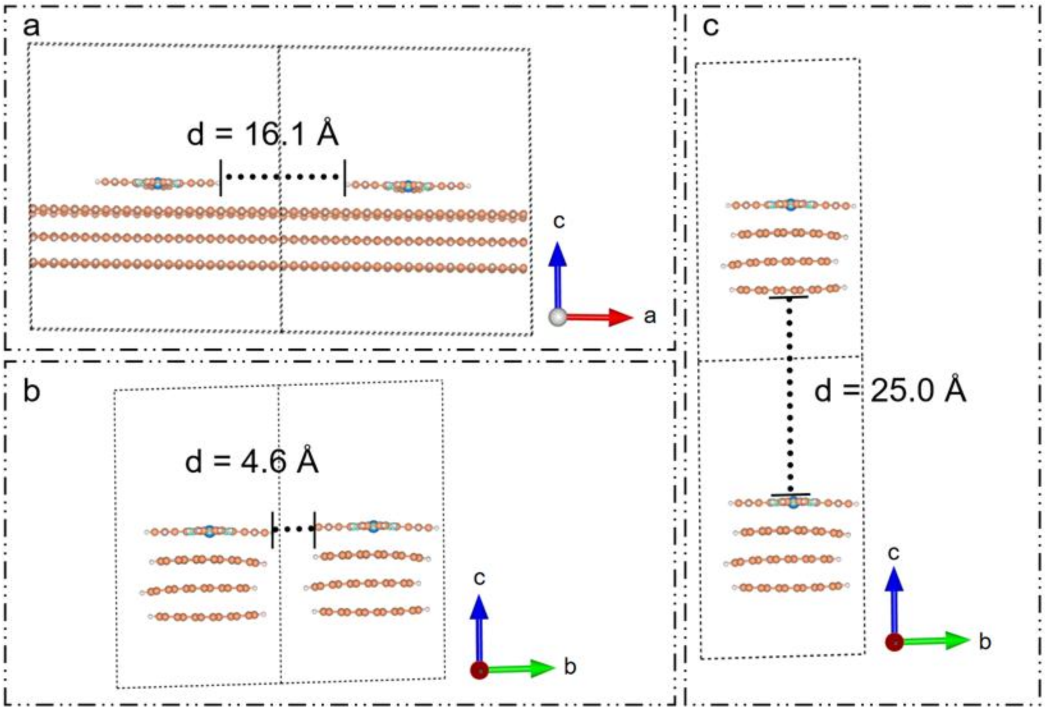

Figure S12. (a) After supercell expansion along the a-axis, the distance between adjacent CoPc molecules is d = 16.1 Å. (b) After expansion along the b-axis, the distance between neighboring CoPc molecules is d = 4.6 Å. (c) After expansion along the c-axis, the separation between CoPc and the graphene substrate is d = 25.2 Å.

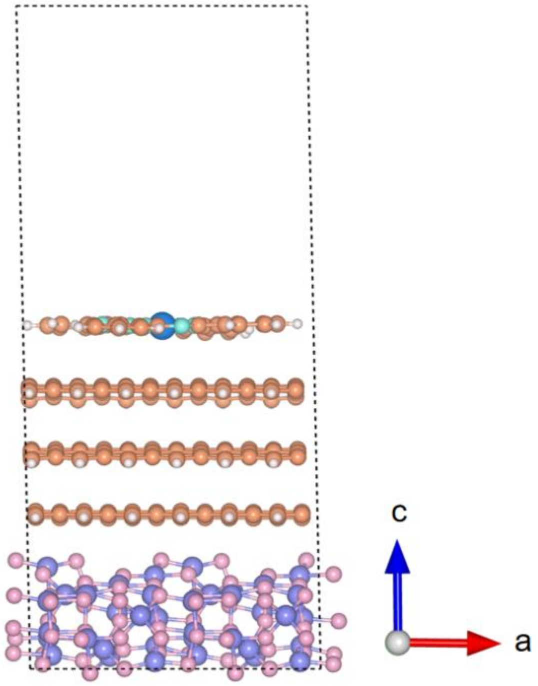

Figure S13. Structural model corresponding to CoPc/Al_2O_3@C-3.

The simulation cell parameters of CoPc/Al_2O_3@C-3 are clarified as follows:

a = 15.55 Å	α = 88.78°
b = 19.49 Å	β = 91.20°
c = 35.46 Å	γ = 90.07°
Volume = 10740.89 Å^3	

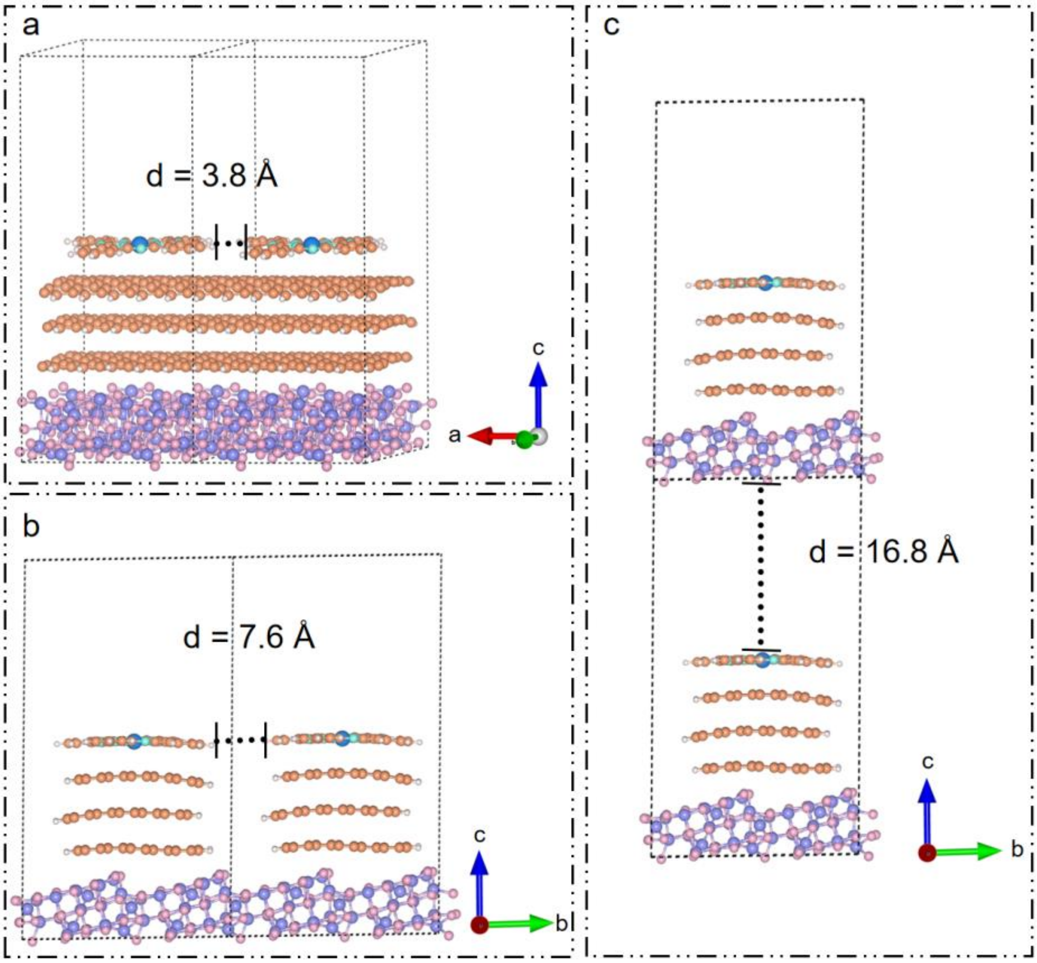

Figure S14. (a) After supercell expansion along the a-axis, the distance between adjacent CoPc molecules is d = 3.8 Å. (b) After expansion along the b-axis, the intermolecular distance between two CoPc molecules is d = 7.6 Å. (c) After expansion along the c-axis, the vertical separation between CoPc and the graphene layer is d = 16.8 Å.

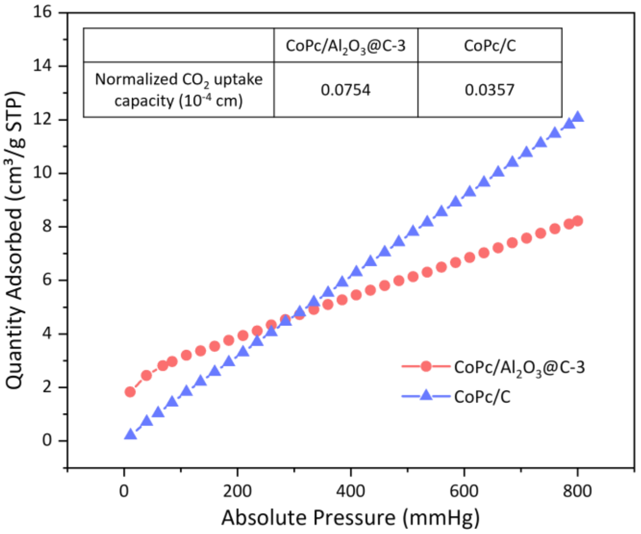

Figure S15. CO_2 uptake of CoPc/Al_2O_3@C-3 and CoPc/C. The table shows the normalized CO_2 uptake capacities of CoPc/Al_2O_3@C-3 and CoPc/C.

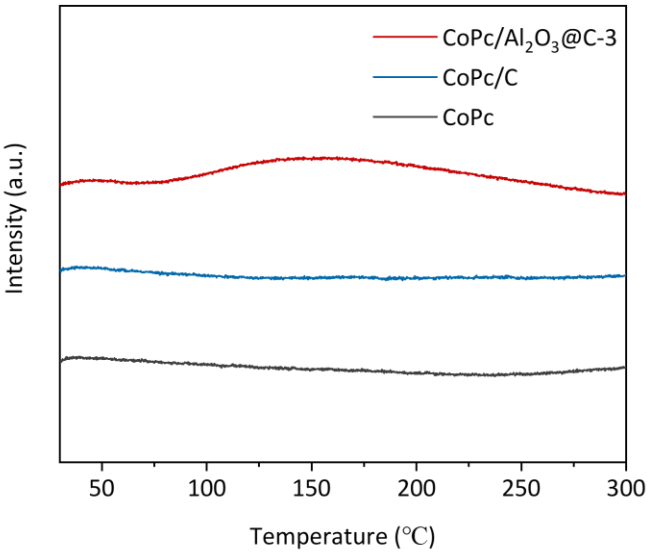

Figure S16. CO_2 TPD curves of CoPc/Al_2O_3@C-3, CoPc/C and CoPc.

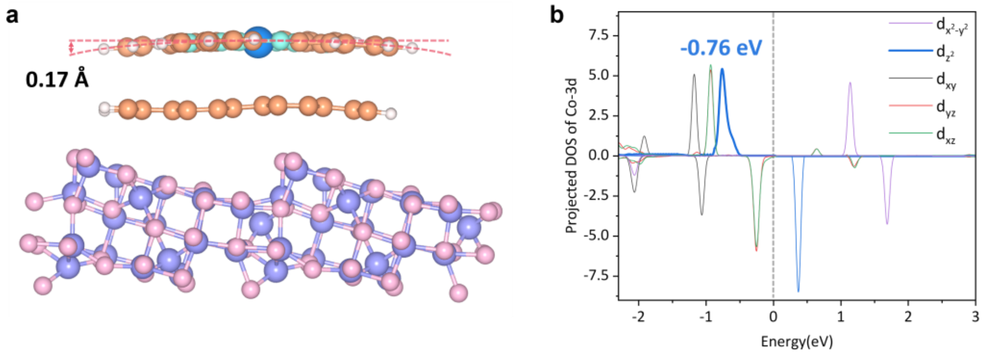

Figure S17. (a) Schematic diagrams of the calculation models (side view) and (b) the calculated PDOS of Co 3d orbitals for CoPc/Al_2O_3@C-1. The labeled values represent the axial distance from the central Co atom to the peripheral H atoms. Co (blue), N (cyan), C (orange), H (white), Al (purple), and O (pink).

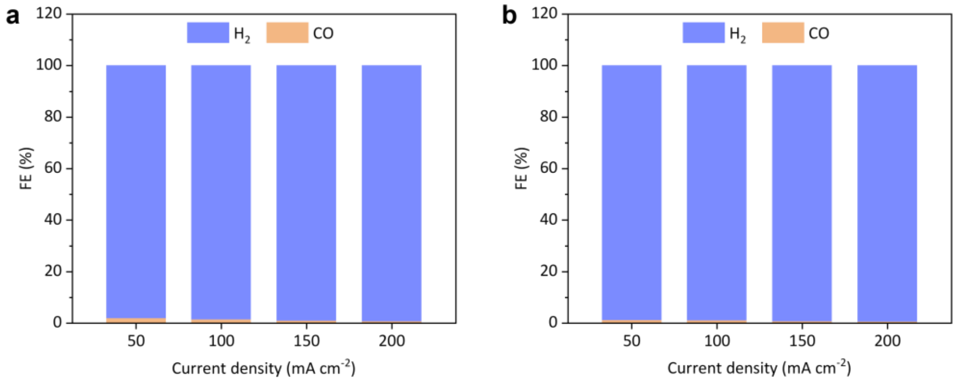

Figure S18. FEs of CO and H_2 at varying current densities for (a) Al_2O_3 and (b) Al_2O_3@C-3.

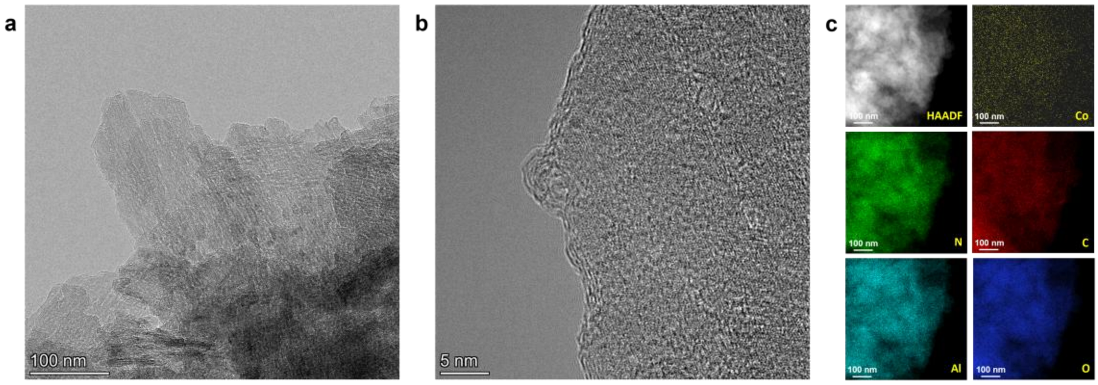

Figure S19. (a) TEM image, (b) HRTEM image and (c) the corresponding EDS elemental mapping images of CoPc/Al_2O_3@C-3 after electrolysis.

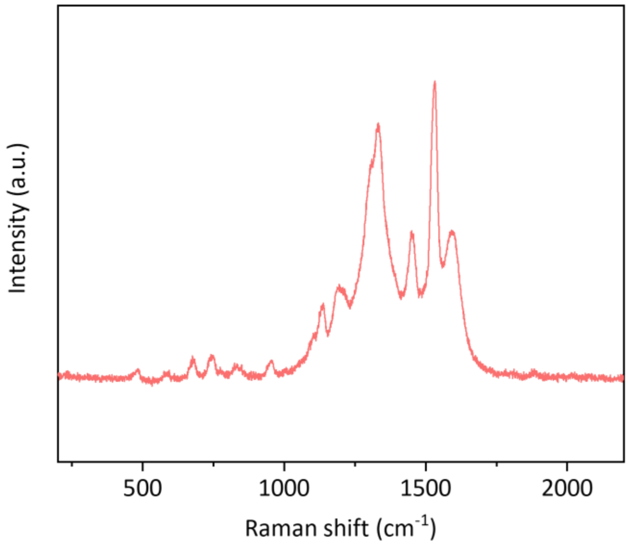

Figure S20. Raman spectrum of CoPc/Al_2O_3@C-3 after electrolysis.

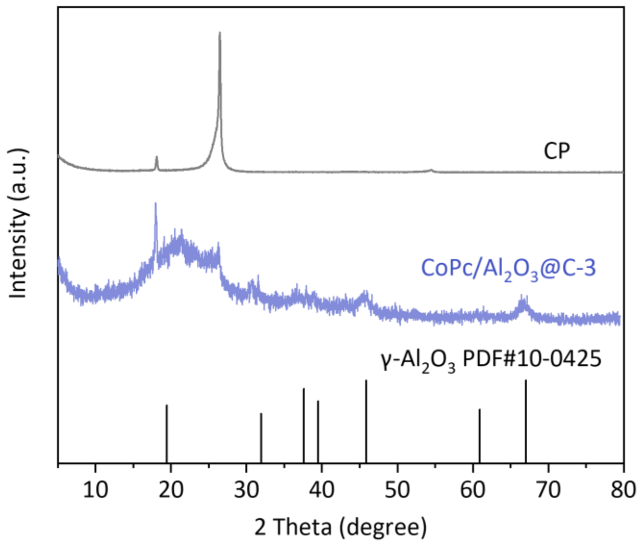

Figure S21. XRD patterns of CoPc/Al_2O_3@C-3 after electrolysis and carbon paper.

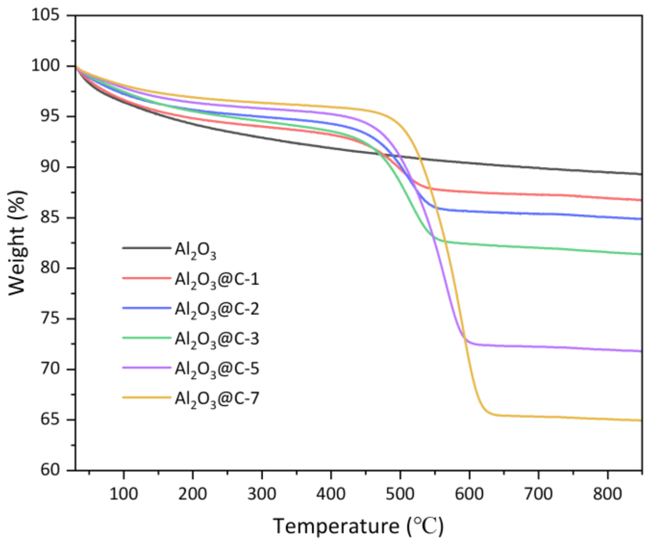

Figure S22. TGA curves of Al_2O_3, Al_2O_3@C-1, Al_2O_3@C-2, Al_2O_3@C-3, Al_2O_3@C-5 and Al_2O_3@C-7.

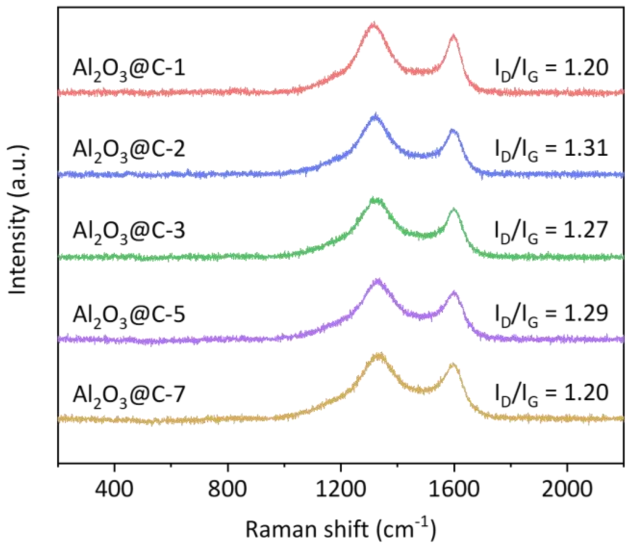

Figure S23. Raman spectra of Al_2O_3@C-1, Al_2O_3@C-2, Al_2O_3@C-3, Al_2O_3@C-5 and Al_2O_3@C-7.

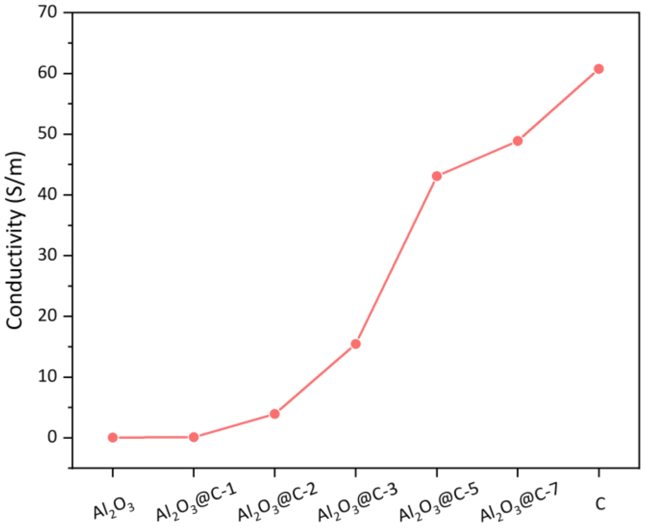

Figure S24. Electric conductivity measured for Al_2O_3, Al_2O_3@C-1, Al_2O_3@C-2, Al_2O_3@C-3, Al_2O_3@C-5 and Al_2O_3@C-7 and C using the four-probe method.

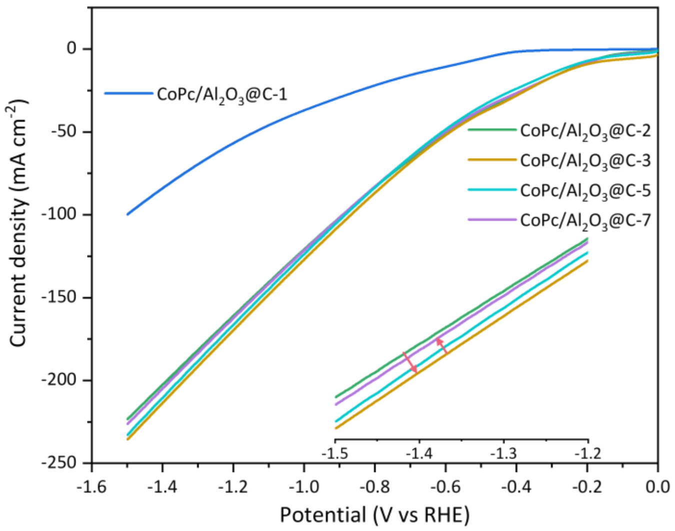

Figure S25. LSVs of CoPc/Al_2O_3@C-1, CoPc/Al_2O_3@C-2, CoPc/Al_2O_3@C-3, CoPc/Al_2O_3@C-5 and CoPc/Al_2O_3@C-7 (inset: the amplified view between -1.2 and -1.5 V).

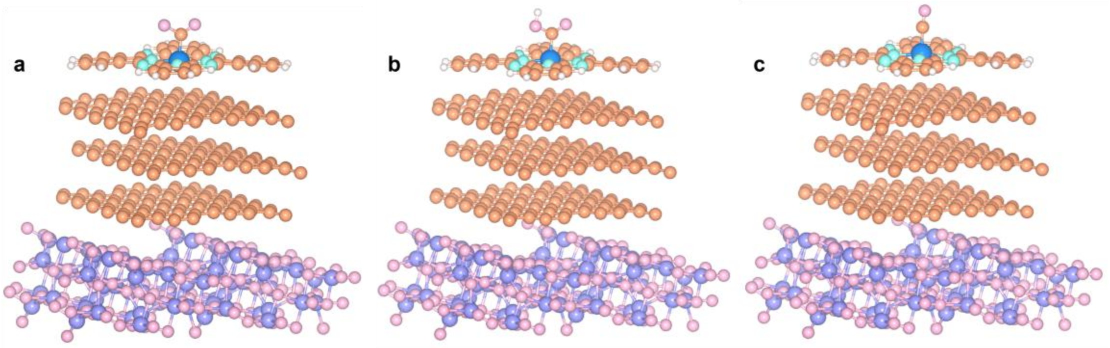

Figure S26. Schematic diagrams of adsorbed *CO_2^-, *COOH and *CO on CoPc/Al_2O_3@C-3. Co (blue), N (cyan), C (orange), H (white), Al (purple), and O (pink).

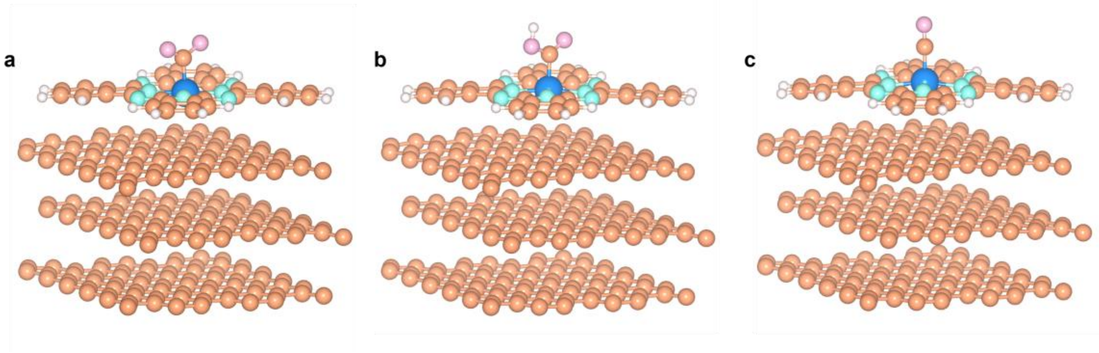

Figure S27. Schematic diagrams of adsorbed $*CO_2^-$, *COOH and *CO on CoPc/C. Co (blue), N (cyan), C (orange), H (white), Al (purple), and O (pink).

Table S1. Weight percentages of Co and Al elements in different samples determined by ICP-AES.

	$CoPc/Al_2O_3$	$CoPc/Al_2O_3$@C-3	CoPc/C
Co	0.238 wt%	0.276 wt%	0.492 wt%
Al	7.876 wt%	3.347 wt%	——

Table S2. Comparisons of eCO_2R performance with different CoPc-based catalysts reported in literature.

Catalysts	Electrolyte	Potential (V)	FE_{CO} (%)	J_{CO} (mA cm^{-2})	Stability	TOF (s^{-1})	Ref
CoPc-P4VP	0.1 M NaH_2PO_4	-1.25	94	2.1	-	5.31	[6]
CoPc@Fe-N-C	0.5 M KOH	-0.84	93	275.6	20 h @ 90 mA cm^{-2}	-	[7]
CoPc2@MWCNTs	1 M KOH	-0.92	94	165	3 h @ 111.6 mA cm^{-2}	3.9	[8]
CoTMAPc/CNT	0.5 M $KHCO_3$	-0.7	95.6	239	15 h @ 30 mA cm^{-2}	102.9	[9]
CoPc/CNT-MD	1 M KOH	-0.9	95	190	38.5 h @ 50 mA cm^{-2}	83.9	[10]
CoPc-CTF	1 M KOH	-1	94.8	378.8	10 h @ 125 mA cm^{-2}	15.58	[11]

Co-CNTs-MW	1 M KOH	-0.7	96	336	24 h @ 100 mA cm^{-2}	7.19	[12]
$CoPc/Mg(OH)_2/NC$	1 M KOH	-0.42	96	285	50 h @ 100 mA cm^{-2}	20.7	[13]
Tc-CoPc	0.1 M $KHCO_3$	-0.585	95	195	24 h @ 100 mA cm^{-2}	29.4	[14]
EtO_8-CoPc/CNP	1 M $KHCO_3$	-	95	340	24 h @ 150 mA cm^{-2}	4.3	[15]
CoPc-H_2Pc	0.5 M $KHCO_3$	-0.65	97	240	10 h @ -0.9 V	27.1	[16]
CoPc/GDY/G	1 M $KHCO_3$	-0.82	99	99	24 h @ 100 mA cm^{-2}	28	[17]
CoPc+P4VP-8	0.1 M $KHCO_3$	-	98	252	35 h @ 100 mA cm^{-2}	-	[18]
1⊃K^+/CNTs/CFP	0.5 M $KHCO_3$	-0.57	96	38	14 h @ -0.63 V	111	[19]
CoPc/C_3N_4/G	0.5 M $KHCO_3$	-	98	67.8	16 h @ -0.8 V	50.5	[20]
CoPc/Al_2O_3@C-3	1 M KOH	-0.48	99.5	388	52 h @ 100 mA cm^{-2}	43	This work

Table S3. Weight percentages of Co and Al elements in different samples determined by ICP-AES.

	$CoPc/Al_2O_3$@C-1	$CoPc/Al_2O_3$@C-2	$CoPc/Al_2O_3$@C-3	$CoPc/Al_2O_3$@C-5	$CoPc/Al_2O_3$@C-7
Co	0.323 wt%	0.303 wt%	0.276 wt%	0.321 wt%	0.332 wt%
Al	5.645 wt%	4.750 wt%	3.347 wt%	3.306 wt%	3.020 wt%

Table S4. Summary of structural and electronic information for CoPc/C-COOH and $CoPc/Al_2O_3$@C-3-COOH.

Sample	∠OCO (deg)	Co-C distance (Å)	Charge of Co (e)	Charge of COOH (e)
CoPc/C	123.68°	2.01	+1.14	-0.64
$CoPc/Al_2O_3$@C-3	121.47°	1.89	+1.05	-0.78

References

1 G. Kresse and J. Furthmiiller, *Comput. Mater. Sci.*, 1996, **6**, 15-50.

2 G. Kresse and J. Furthmu¨ller, *Phys. Rev. B*, 1996, **54**, 11169-11186.

3 S. Grimme, *WIREs Computational Molecular Science*, 2011, **1**, 211-228.

4 S. Grimme, S. Ehrlich and L. Goerigk, *J. Comput. Chem.*, 2011, **32**, 1456-1465.

5 E. Sanville, S. D. Kenny, R. Smith and G. Henkelman, *J. Comput. Chem.*, 2007, **28**, 899-908.

6 Y. Liu and C. C. L. McCrory, *Nat. Commun.*, 2019, **10**, 1683.

7 L. Lin, H. Li, C. Yan, H. Li, R. Si, M. Li, J. Xiao, G. Wang and X. Bao, *Adv. Mater.*, 2019, **31**, 1903470.

8 M. Wang, K. Torbensen, D. Salvatore, S. Ren, D. Joulié, F. Dumoulin, D. Mendoza, B. Lassalle-Kaiser, U. Işci, C. P. Berlinguette and M. Robert, *Nat. Commun.*, 2019, **10**, 3602.

9 J. Su, J.-J. Zhang, J. Chen, Y. Song, L. Huang, M. Zhu, B. I. Yakobson, B. Z. Tang and R. Ye, *Energy Environ. Sci.*, 2021, **14**, 483-492.

10 X. Wu, J. W. Sun, P. F. Liu, J. Y. Zhao, Y. Liu, L. Guo, S. Dai, H. G. Yang and H. Zhao, *Adv. Funct. Mater.*, 2021, **32**, 2107301.

11 Q. Wu, D. H. Si, J. Liang, Y. B. Huang and R. Cao, *Appl. Catal., B*, 2023, **333**, 122803.
12 J. W. Sun, X. Wu, P. F. Liu, J. Chen, Y. Liu, Z. X. Lou, J. Y. Zhao, H. Y. Yuan, A. Chen, X. L. Wang, M. Zhu, S. Dai and H. G. Yang, *Nat. Commun.*, 2023, **14**, 1599.
13 F. Lyu, B. Ma, X. Xie, D. Song, Y. Lian, H. Yang, W. Hua, H. Sun, J. Zhong, Z. Deng, T. Cheng and Y. Peng, *Adv. Funct. Mater.*, 2023, **33**, 2214609.
14 S. Zhou, L. J. Zhang, L. Zhu, C. H. Tung and L. Z. Wu, *Adv. Mater.*, 2023, **35**, 2300923.
15 S. Ren, E. W. Lees, C. Hunt, A. Jewlal, Y. Kim, Z. Zhang, B. A. W. Mowbray, A. G. Fink, L. Melo, E. R. Grant and C. P. Berlinguette, *J. Am. Chem. Soc.*, 2023, **145**, 4414-4420.
16 R. Wang, X. Wang, W. Weng, Y. Yao, P. Kidkhunthod, C. Wang, Y. Hou and J. Guo, *Angew. Chem., Int. Ed.*, 2021, **61**, e202115503.
17 H. Gu, L. Zhong, G. Shi, J. Li, K. Yu, J. Li, S. Zhang, C. Zhu, S. Chen, C. Yang, Y. Kong, C. Chen, S. Li, J. Zhang and L. Zhang, *J. Am. Chem. Soc.*, 2021, **143**, 8679-8688.
18 B. Chen, Y. Rong, X. Li, J. Sang, P. Wei, Q. An, D. Gao and G. Wang, *ACS Energy Lett.*, 2024, **9**, 911-918.
19 L. Zhu, Y. X. Wang, L. J. Chen, J. Li, S. Zhou, Q. Q. Yang, X. Z. Wang, C. H. Tung and L. Z. Wu, *Angew. Chem., Int. Ed.*, 2024, **64**, e202418156.
20 Z. Wang, J. Yang, Z. Song, M. Lu, W. Wang, Z. Ren and Z. Chen, *ACS Catal.*, 2024, **14**, 8138-8147.
